# Assessing Bacterial Diversity in the Rhizosphere of *Thymus zygis* Growing in the Sierra Nevada National Park (Spain) through Culture-Dependent and Independent Approaches

**DOI:** 10.1371/journal.pone.0146558

**Published:** 2016-01-07

**Authors:** Javier Pascual, Silvia Blanco, Marina García-López, Adela García-Salamanca, Sergey A. Bursakov, Olga Genilloud, Gerald F. Bills, Juan L. Ramos, Pieter van Dillewijn

**Affiliations:** 1 Estación Experimental del Zaidín, Spanish National Research Council (CSIC), Granada, Spain; 2 MEDINA Foundation, Centre of Excellence for Innovative Medicines Research, Granada, Spain; Graz University of Technology (TU Graz), AUSTRIA

## Abstract

Little is known of the bacterial communities associated with the rhizosphere of wild plant species found in natural settings. The rhizosphere bacterial community associated with wild thyme, *Thymus zygis* L., plants was analyzed using cultivation, the creation of a near-full length 16S rRNA gene clone library and 454 amplicon pyrosequencing. The bacterial community was dominated by *Proteobacteria* (mostly *Alphaproteobacteria* and *Betaproteobacteria*), *Actinobacteria*, *Acidobacteria*, and *Gemmatimonadetes*. Although each approach gave a different perspective of the bacterial community, all classes/subclasses detected in the clone library and the cultured bacteria could be found in the pyrosequencing datasets. However, an exception caused by inconclusive taxonomic identification as a consequence of the short read length of pyrotags together with the detection of singleton sequences which corresponded to bacterial strains cultivated from the same sample highlight limitations and considerations which should be taken into account when analysing and interpreting amplicon datasets. Amplicon pyrosequencing of replicate rhizosphere soil samples taken a year later permit the definition of the core microbiome associated with *Thymus zygis* plants. Abundant bacterial families and predicted functional profiles of the core microbiome suggest that the main drivers of the bacterial community in the *Thymus zygis* rhizosphere are related to the nutrients originating from the plant root and to their participation in biogeochemical cycles thereby creating an intricate relationship with this aromatic plant to allow for a feedback ecological benefit.

## Introduction

The rhizosphere, or the soil under the influence of plant roots [1], is considered one of the most diverse microbial habitats with respect to species richness and community size [2]. The rhizosphere bacterial community can affect plant health by playing important roles in nutrient acquisition, protection against adverse environmental conditions and plant pathogens, and in plant growth promotion through, for instance, the production of plant hormones [2–4]. Within the soil, rhizosphere bacteria also participate in soil formation and the biogeochemical cycling of carbon, nitrogen, phosphorus, and other elements [5]. Moreover, these microorganisms may also be involved in other important processes including the removal or degradation of toxic and/or recalcitrant organic contaminants [6,7].

The importance of the rhizosphere has led to widespread interest in understanding the diversity and function of the microbial communities which make up this microbiome. As a result, most studies regarding rhizosphere microbial diversity have centred on crop plants or model plants such as *Arabidopsis* spp. However, although some progress is being made, the rhizosphere of non-cultivated plant species remains largely unknown [5]. Members of the genera *Thymus* constitute aromatic plants typical of Mediterranean shrublands which may participate in vegetation succession [8] and as seeders for re-vegetation after forest fires [9]. In addition, the essential oils and volatile compounds derived from thyme species are valuable for the pharmaceutical, food and perfume industries due to their medicinal, anti-oxidant, fragrance and culinary properties. Within this context, the wild thyme species *Thymus zygis* L. are particularly interesting as a source for essential oils with high contents of thymol, a monoterpene phenol with antifungal and antibacterial activities [10]. Nonetheless, aside from studies of the arbuscular mycorrhizal communities associated with this plant [11] little is known of the diversity of the microbial communities present in its rhizosphere.

The aim of the present study was to analyze the bacterial diversity naturally present in the rhizosphere associated with wild *Thymus zygis* L. Since the assembly of microbial communities in the rhizosphere can be affected by human activities such as the input of fertilizers and pesticides [5], a pristine site in the Sierra Nevada National Park located in the southeast of the Iberian Peninsula was used as the study site to reduce possible anthropogenic effects. To thoroughly study these rhizosphere bacterial communities, data obtained through cultivation methods, a near-full length 16S rRNA gene clone library and 454 amplicon pyrosequencing were integrated and compared. This together with further amplicon sequencing has revealed the core rhizosphere soil bacterial community associated with *Thymus zygis*. Altogether, these results are discussed within the context of the technical constraints of the methodologies used and the possible functional roles of the bacterial communities found with regard to their adaption to this rhizosphere niche.

## Material and Methods

### Rhizosphere soil sampling, processing and physicochemical characteristics

Three apparently healthy *Thymus zygis* L. plants were collected at an elevation of approximately 2000 m previous to their seasonal flowering period in the Sierra Nevada National Park, Granada, Spain (36° 57´ 55.4´´N 3° 20´ 19.4´´W) in April 2010 and again at the same location in May 2011. Individually growing, mature (woody, 11–15 cm tall) plants were selected within a distance of 1 to 13 m from each other. Permits for sampling were obtained from the authorities of the Sierra Nevada National Park whom likewise facilitated access to protected areas. From each individual plant, roots with soil were taken and stored in sterile plastic bags for transport to the laboratory where samples were further processed (maximum time between sampling and processing was 12 h).

To obtain the rhizosphere soil sample, roots from each plant were separated from the shoot and soil not adhering to the roots removed. For the sampling period of April 2010, roots from three plants were pooled while for the sampling period of May 2011 roots from each of three different plants were treated separately. Treatment consisted of washing the root material twice with 100 mL sterile PBS at room temperature by vigorous shaking for 5–10 minutes in a closed container. After removing roots, the resulting slurry from both washes were mixed in a centrifuge container and centrifuged for 15 minutes at 8000 g. Almost all of the supernatant was removed and the remaining rhizosphere soil was used as the source for rhizosphere bacterial isolates and metagenomic DNA. The physicochemical characteristics of the soil in which the plants were growing in 2010 were determined by the Andalusian soil analysis laboratory (Laboratorio Agroalimentario de Atarfe, Granada, Spain) using standard international methods. The soil of the sampling site had a sandy loam texture, nearly neutral pH, an electrical conductivity (salinity) of 0.04 mmhos cm^-1^, 1.24% humic matter, 1.6% carbonates and a total nitrogen concentration of 0.082% (S1 Table).

### Isolation, cultivation and molecular identification of cultivable bacteria

Rhizosphere soil from the samples taken in 2010 (1 g wet weight) was dispersed in 100 mL of sterile diluent (VL70 medium without added growth substrate or vitamins) in 250-mL Erlenmeyer flasks by stirring for 30 min and serial dilutions spread (five replicates at each dilution) onto the surface of the isolation medium with sterile glass spreading rods. The medium used for the isolations was gellan gum-solidified VL70 containing as growth substrates a mixture of peptone-casein, 0.025% (w/v) [12,13]. The culture medium was supplemented with 0.1% (w/v) cycloheximide to inhibit fungi. Isolation plates were incubated for six weeks at 18°C and 60% relative humidity in the dark. After incubation, 148 bacterial colonies were randomly picked and transferred to new Petri dishes filled with R2A medium (Becton-Dickinson, Sparks, MD, USA), a relatively low nutrient medium, albeit more complex than VL70 medium. Pure cultures were frozen in glycerol 20% (v/v) at -80°C for long-term storage.

The total number of viable bacterial cells per gram of wet rhizosphere soil was determined by microscopic counts of preparations stained using the LIVE/DEAD *Bac*Light bacterial viability kit (Molecular Probes Inc., Eugene, Oreg, USA) according to manufacturer's instructions. The colony forming units (CFUs) per g of wet rhizosphere soil was also determined after six weeks of incubation. Cultivability was calculated as the percentage of CFUs recovered from the total number of viable bacterial cells. The taxonomic identities of isolates were assigned by 16S rRNA gene sequence analysis. Bacterial genomic DNAs were extracted by microwave lysis [14] by suspending a few colonies from each strain in 750 μL of MilliQ water in a microcentrifuge tube and irradiation at maximum power in a microwave oven in three sessions of alternating 45 s pulses with 30 s recovery intervals. For recalcitrant bacteria, DNAs were alternatively isolated by using a GeneJET Genomic DNA Purification Kit (Thermo Fisher Scientific Inc., Waltham, MA, USA). For nearly full-length amplification of the 16S rRNA gene, the primer pair FD1 (5´-AGAGTTTGATCCTGGCTCAG-3´) and RP2 (5´- ACGGCTACCTTGTTACGACTT-3´) was used [15]. PCR mixtures were composed of 5.0 μL PCR buffer (10×), 4.0 μL MgCl_2_ (final concentration 2 mM), 1.0 μL dNTPs (10 mM each), 1.0 μL each forward and reverse primers (10 mM), 0.3 μL Taq polymerase (5 U μL^-1^ Qbiogene) and 5.0 μL DNA solution in a total volume of 50 μL. The thermal cycling program was described previously [16]. PCR products were purified and sequenced using the above primer pair and the internal primers 926F (5´-AAACTYAAAKGAATTGACGG-3´) and 1100R (5´-GGGTTGCGCTCGTTG-3´) at Secugen S.L. (Madrid, Spain).

### Metagenomic DNA extraction, construction of a 16S rRNA gene clone library and 454 pyrosequencing

Metagenomic DNA was extracted from all rhizosphere soil samples using the Fast DNA Spin Kit for Soil (MP Biomedicals, LLC., Solon, OH, USA) following the manufacturer´s instructions. DNA was further purified by separation on 1.25% agarose gels amended with 2% polyvinylpyrrolidone and isolation from the excised bands using QIAQUICK Gel Extraction Kit (Qiagen, Hilden, Germany). DNA integrity was checked by agarose gel electrophoresis and quantified spectrophotometrically.

For the 16S rRNA gene clone library, approximately 1450 bp long amplification products were obtained using universal primers GM3F (5’-AGAGTTTGATCMTGGC-3’) and GM4R (5’-TACCTTGTTACGACTT-3’) [17]. Metagenomic DNA obtained from the pooled rhizosphere soil taken in 2010 was amplified in 50-μL reaction volumes with 2.5 U *Taq* DNA polymerase (Roche Molecular Biochemicals, Indianapolis, IN, USA), 20 ng of metagenomic DNA, 250 μM of each dNTP, 1.5 mM MgCl_2_, 200 nM of each primer and the appropriate buffer supplied by the manufacturer. The amplification program was as follows: 1 cycle of 5 min at 95°C; 30 cycles of 45 s at 95°C, 45 s at 45°C, and 2 min at 72°C; and, finally, 1 cycle of 10 min at 72°C. PCR were run in duplicate, and the resulting amplicons were further pooled prior to purification by running the PCR amplicons on 1% (w/v) agarose gels. Amplicons were excised and purifying using the QIAQUICK Gel Extraction Kit (Qiagen, Hilden, Germany). The resulting DNA was ligated in triplicate into the pGEM-T plasmid vector (Promega Corp., Madison, WI, USA) and subsequently transformed into competent cells of *E*. *coli* strain DH5α. Using an automated colony picking robot (Qpix2, GENETIX), from the approximately 8000, white transformed colonies growing on LB plates supplemented with 50 μg mL^-1^ (w/v) ampicillin and 10 μg mL^-1^ X-gal, 384 (approximately 5%) were randomly picked and sequenced using the M13 forward (5’-GACGTTGTAAAACGACGGCCAG-3’) and M13 reverse (5’-GAGGAAACAGCTATGACCATG-3’) primers by the sequencing service provided by Secugen (Madrid, Spain). Sequences belonging to the 16S rRNA gene clone library were analyzed systematically. Nucleotide sequences were manually checked, forward and reverse complementary sequences were assembled and trimmed of vector sequences using DNA Baser Sequence Assembler software (Heracle BioSoft S.R.L., Pitesti, Romania). Chimera sequences were detected and further removed by the web-based version of the USEARCH 6.0 chimera detection tool [18].

For pyrosequencing, the V123 region of the 16S rRNA gene was amplified from the pooled rhizosphere metagenomic DNA sample of 2010 or the separate DNA samples from 2011 using the eubacterial primers at *E*. *coli* position 8 (5´-AGAGTTTGATCMTGGCTCAG-3´) and position 532 (5´-TACCGCGGCKGCTGGC-3´) or 8 (5´- TCAGAGTTTGATCCTGGCTCAG-3´) and position 532 (5´- CACCGCGGCKGCTGGCAC-3´) [19], respectively, appended with the 454 A or B fusion sequence together with a 4 bp key tag and 10 bp barcode. PCR were performed with 20 ng of metagenomic DNA, 200 μM of each of the four dNTPs, 1.5 mM of MgCl_2_, 200 nM of each primer, 2.5 U of Taq DNA polymerase (Roche), and the appropriate buffer supplied by the manufacturer. The amplification program used was as follows: 1 cycle of 5 min at 95°C; 35 cycles of 45 s at 95°C, 45 s at 55°C, and 2 min at 72°C; and, finally, 1 cycle of 10 min at 72°C. PCR were repeated in quintuplicate and pooled prior to purification by running the PCR amplicons on 1% (w/v) agarose gels. The bands containing DNA from the amplified products were excised and purified using the QIAQUICK Gel Extraction Kit (Qiagen, Hilden, Germany). Subsequently, amplicons were quantified using picogreen (Invitrogen) and submitted to the pyrosequencing services offered by CITIUS-Center for Research, Innovation and Technology (University of Seville, Spain) (for the 2010 amplicon library) or to Macrogen (Seoul, South Korea) (for the separate 2011 amplicon libraries) where in either case EmPCR was performed prior to bidirectional pyrosequencing with the Roche GS FLX Titanium instrument. Partial 16S rRNA gene sequences were trimmed according to the initial data processing step in the RDP pyrosequencing pipeline [20] with default parameters (max number of N's = 0, minimum average quality score = 20 and minimum sequence length = 350 bp). Chimera sequences were detected and removed in the same fashion as the 16S rRNA gene sequences retrieved from the clone library.

### Bacterial diversity, richness and taxonomic distribution of taxa

For most of the 16S rRNA gene sequence analysis, the online Ribosomal Database Project (RDP) release 11, update 3 was used [20,21]. For taxonomic-based analysis the RDP Classifier tool was used at confidence level of 80% for all six datasets. In the case of pyrosequencing datasets, the 16S rRNA gene copy number of each taxon was corrected in order to obtain more accurate abundance estimates [22]. The similarities of cultured bacteria and clone library sequences with closest type strains were determined using the SeqMatch tool of RDP [20]. After independently aligning each sequence dataset with the RDP Infernal aligner, a fast secondary-structure aware aligning algorithm [23,24], OTUs were defined at the species level (3% sequence divergence) [25] using the Complete Linkage Clustering tool of RDP [20]. For Beta diversity analyses between pyrosequencing datasets (2010 vs. 2011), operational taxonomic units (OTUs) were identified using the open reference OTU picking pipeline implemented in QIIME V1.8.0 [26]. For the OTU picking, the algoritm usearch61 with a 97% clustering identity and the Greengenes database release 13.8 were used. For multivariable analysis weighted Unifrac distances were calculated and visualized by Principal Coordinate Analysis (PCoA). For the prediction of functional and metabolic profiles of the bacterial community based on the 16S rRNA gene sequences from each dataset the newly developed open-source R package Tax4Fun was used [27] after data processing with QIIME [26] and the SILVA database 119 [28] as required for this tool. The taxonomic profiles were normalized by the 16S rRNA gene copy number and the functional reference profiles were computed based on 400 bp reads. Box-plots were generated using ggplot2 package in R (http://had.co.nz/ggplot2/).

All 16S rRNA gene sequences were submitted to GenBank/EMBL/DDBJ under the accession numbers JX840944-JX841091 for cultured bacteria and JX114334-JX114519 for the 16S rRNA gene clone library. The pyrosequencing data were submitted to the SRA database under accession numbers SRR957690 and SRR2924985.

## Results

### Cultured bacterial community

**Fig 1:**
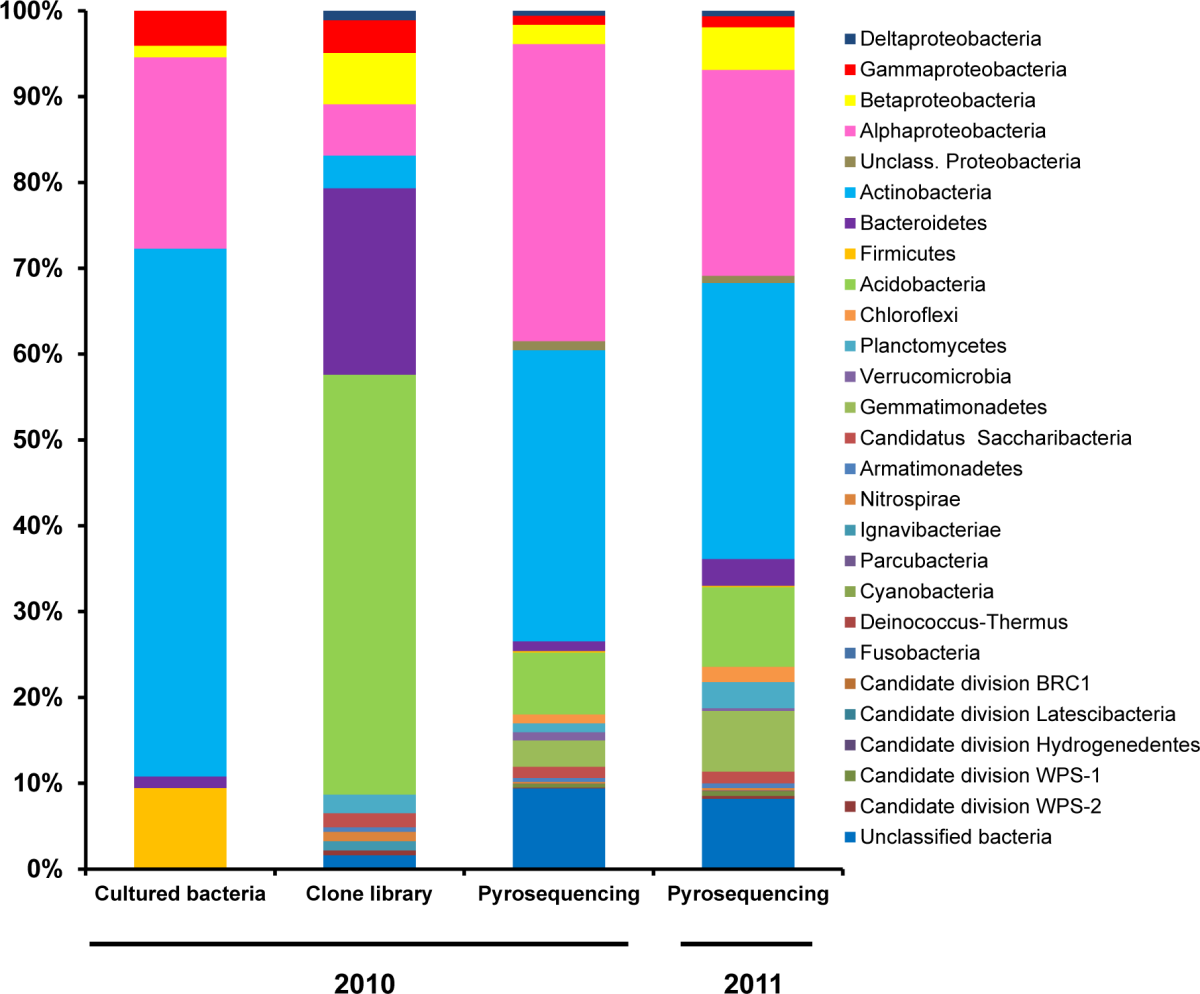
Relative abundance of the different bacterial phyla and proteobacterial classes identified through culture-dependent and culture independent (clone library and 454 pyrosequencing) methodologies targeting the 16S rRNA gene. Bacterial sequences were classified with the RDP classifier tool (Release 11, Update 3), selecting 80% as the confidence threshold and adjusting the copy number of 16S rRNA operons in the case of the pyrosequencing.

Prior to cultivating bacteria from the *Thymus zygis* rhizosphere soil collected in 2010, the total number of viable bacterial cells per g rhizosphere soil was determined microscopically to be 1.6 × 10^9^. The average number of CFUs obtained per g rhizosphere soil on VL70 medium containing a mixture of peptone-casein was 9.7 × 10^6^ ± 1.9 × 10^6^ after six weeks of incubation. The cultivability, expressed as the percentage of CFUs recovered compared to the total number of viable bacterial cells, was 0.60% of the total. A total of 148 colonies were randomly isolated and successfully cultured *in vitro*. Identification using 16S rRNA gene sequencing resulted in a dataset containing high-quality partial and nearly complete 16S rRNA gene sequences (lengths between 531 to 1509 bp) depending on the taxonomic interest of each specific strain (S2 Table). Except for two and three strains which remained unclassified at the family and genus level respectively, all of the cultured bacteria could be classified into 4 different phyla, 7 bacterial classes, 11 orders, 26 families and 37 genera (S1 Fig). Four point seven percent of the isolates shared less than 97% to as low as 95.5% 16S rRNA gene similarity with their closest type strains which corresponded to the genera *Methylobacterium* (*Alphaproteobacteria*) and *Nocardioides-Streptomyces* (*Actinobacteria*) (S2 Table). Within the cultured bacteria collection, most (61.5%) belong to the phylum *Actinobacteria*, almost all of which fall into the subclass *Actinobacteridae* and only 1 isolate into the subclass *Rubrobacteridae*. Isolates belonging to the phylum *Proteobacteria* fell within three different classes: *Alphaproteobacteria* (22.3%), *Gammaproteobacteria* (4.1%) and *Betaproteobacteria* (1.4%). The next most common phyla were *Firmicutes* (9.5%), of which all were identified as *Bacilli*, and the phylum *Bacteroidetes* (1.4%) which were equitably classified as *Sphingobacteriia* or *Flavobacteriia* (Fig 1).

### 16S rRNA gene clone library

A total of 384 clones (approximately 5% of all of the transformed colonies obtained) were sequenced from the 16S rRNA gene clone library generated from the rhizosphere soil DNA of the 2010 pooled sample. After quality filtering, only 184 high quality near-full length and non-chimeric reads could be assembled and used for downstream analysis. Sequence lengths ranged from 1077 to 1092 bp. The nucleotide sequence of the cloned fragments of the 16S rRNA gene could be classified into 10 bacterial phyla, 15 classes, 15 orders, 23 families and 37 genera (S1 Fig). However, 3, 3, 12, 3, and 11 clones could not be classified at the phylum, class, order, family or genus taxonomic ranks, respectively. Of the classified clone sequences, 48.9% could be affiliated to the phyla *Acidobacteria*, 21.7% to *Bacteroidetes*, 16.8% to *Proteobacteria*, and 3.8% to *Actinobacteria*. Other minority phyla were the “*candidatus* Saccharibacteria” (1.6%), *Planctomycetes* (2.2%), *Nitrospirae* (1.1%), *Ignavibacteriae* (1.1%), *Armatimonadetes* (0.5%) and candidate division WPS-2 (0.5%). The *Acidobacteria* clones fell into four classes: Gp1, Gp4, Gp6 and Gp7, with Gp6 and Gp4 being the most numerous. The *Bacteroidetes* classes observed belonged to *Cytophagia*, *Flavobacteriia* and *Sphingobacteriia*, with the latter as the most abundant. Among the *Proteobacteria*, *Alphaproteobacteria* and *Betaproteobacteria* were the most predominant classes followed by *Gammaproteobacteria* and *Deltaproteobacteria*, respectively. Within the phylum *Actinobacteria*, clones were affiliated to the classes *Acidimicrobiae* and *Rubrobacteridae* (Fig 1). Out of 184 clones, 13, 89 and 55 shared similarity values between 97–95%, 97–85% and <85% with their respective closest type strain (S3 Table). These results show the high degree of taxonomic novelty present in this bacterial community, mainly at higher taxonomic ranks.

### 16S rRNA gene amplicon pyrosequencing

The pyrosequencing-based analysis of the V123 region of the 16S rRNA gene from metagenomic DNA from the 2010 rhizosphere pooled sample resulted in the recovery of 17,948 high quality non-chimeric sequences from the 27,909 reads initially included in the pipeline. The average read length was 497 ± 18.05 bp. After the taxonomic normalization by the 16S rRNA gene copy number, a total number of 8751 sequences were retained. Pyrosequencing revealed the presence of 16 phyla or candidate divisions, 39 bacterial classes, 44 bacterial orders, 96 families or 250 different genera in the rhizosphere soil sample (S1 Fig). Of all the sequences, 9.4% of the pyrotags could not be classified at the phylum or candidate division. Only one read could be assigned to chloroplasts (*Eukaryota*) and was not analyzed further. The most common phyla were *Proteobacteria* (39.5% of all pyrotags), *Actinobacteria* (33.9%), *Acidobacteria* (7.2%), *Gemmatimonadetes* (3.1%), “*candidatus* Saccharibacteria” (1.3%), *Bacteroidetes* (1.1%), *Planctomycetes* (1.0%), *Verrucomicrobia* (1.0%) and *Chloroflexi* (1.0%). Representatives of the phyla/candidate divisions WPS-1, *Armatimonadetes*, *Nitrospirae* and *Firmicutes*, were detected below 1% each, and WPS-2, *Parcubacteria* and *Hydrogenedentes* below 0.1%. Among the proteobacterias, the *Alphaproteobacteria* was the largest class (34.6% of all pyrotags), followed by *Betaproteobacteria*, *Gammaproteobacteria* and *Deltaproteobacteria* which accounted for 2.2%, 1.0% and 0.6%, respectively of the sequences. A group of unclassified *Proteobacteria* was also detected (1.0%). Within *Actinobacteria*, the subclass *Actinobacteridae* was the most numerous (19.6%), followed by *Rubrobacteridae* (10.4%) and *Acidimicrobidae* (1.4%). Less than 0.1% of the reads belonged to class *Thermoleophilia*, and some (1.2%) of the *Actinobacteria* sequences remained unclassified. Representatives of ten classes/subdivisions within the phylum *Acidobacteria* were found, with Gp6 (2.2%), Gp16 (1.7%) and Gp4 (1.4%) as the classes with the most reads. All the *Gemmatimonadetes* sequences fall into the only class with taxonomic validation described so far for this phylum, the class *Gemmatimonadetes* (http://www.bacterio.net/). Sequences belonging to the phylum *Bacteroidetes* could be grouped into the classes *Sphingobacteriia* (0.7%), *Cytophagia* (0.2%) and *Flavobacteriia* (0.01%), whereas 0.2% remained as unclassified *Bacteroidetes*. Sequences belonging to *Planctomycetes* harboured mainly representatives of the class *Planctomycetacia* and also a few *Phycisphaerae*. Finally, sequences belonging to *Chloroflexi* could be assigned into five classes, with the *Caldilineae*, *Thermomicrobia* and *Chloroflexia* as the most abundant classes (Fig 1).

### Comparison among the three approaches used for the 2010 rhizosphere sample

**Fig 2:**
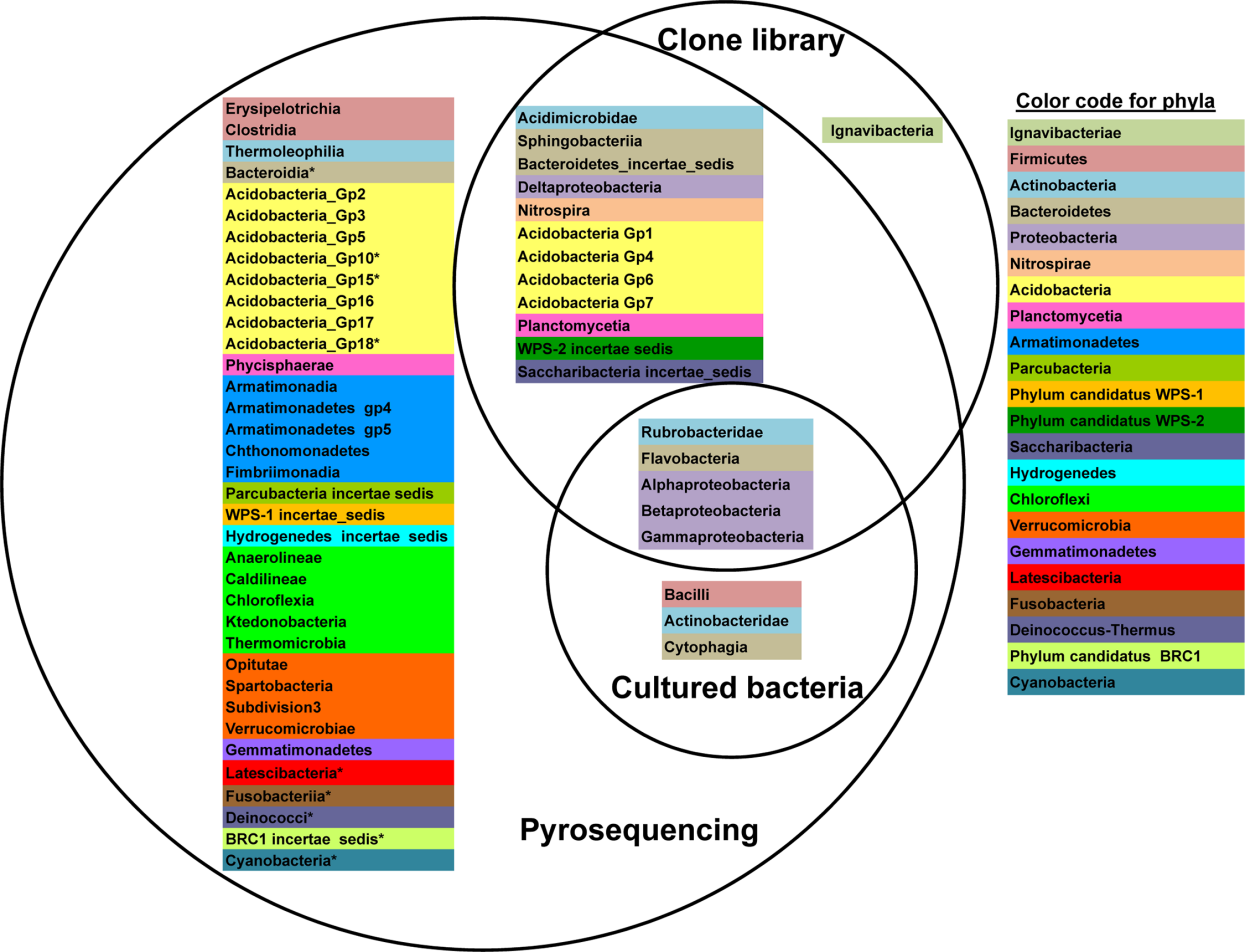
Venn diagram at the class level. Classification at the rank of class/subclass showing shared and unique taxa identified with each approach. Bacterial classes or in the case of *Actinobacteria* subclasses which belong to the same phylum are highlighted with the same color. Asterisks indicate those phyla detected in the 2011 pyrosequencing datasets but not in the 2010 pyrosequencing dataset.

A comparison to determine to what degree the sequences retrieved among the three approaches from the same pooled rhizosphere soil of 2010 were shared at the class/subclass level is represented as a Venn diagram (Fig 2). Of the 17 phyla/ candidate divisions recorded globally in the *Thymus zygis* rhizosphere soil in 2010, only proteobacterias (*Alphaproteobacteria*, *Betaproteobacteria* and *Gammaproteobacteria*), actinobacterias belonging to the subclass *Rubrobacteridae* and *Bacteroidetes* class *Flavobacteriia* were detected by all three approaches. No taxa were exclusively recovered with the culture-dependent approach. However, members of *Bacilli*, *Actinobacteridae* and *Cytophagia* which were successfully cultured were also detected by pyrosequencing but not in the clone library dataset. In fact, no sequences classified as belonging to the *Firmicutes* phylum were detected in the clone library dataset. More surprisingly, representatives of the lineage *Ignavibacteriae* appeared to be unique to the clone library. However, when the sequences of the clones classified as *Ignavibacteriae* (clones SNNP_2012_60 and SNNP_2012_78) are trimmed to contain only regions V123 (500 bp in length) as if they were pyrotags, the taxonomic assignment changed to become unclassifiable at the phylum level. Therefore, any possible representatives of *Ignavibacteriae* present in the pyrosequencing dataset would likewise be identified as unclassified bacteria. Of the remaining 12 phyla/ candidate divisions, *Parcubacteria*, *Hydrogenedetes*, *Chloroflexi*, *Verrucomicrobia*, *Gemmatimonadetes* and candidate division WPS-1 lineages were only detected in the pyrosequencing reads.

Since the contents of current databases will introduce bias in the number of species and genera classified into each phyla/class, the alpha-diversity parameters of each approach were calculated as Operational Taxonomic Units (OTUs) at 97% sequence similarity. The relative richness, calculated as the number of OTUs observed with regard to the total number of sequences within of each dataset, was 60.1%, 75.0% and 47.0% for cultured bacteria, clone library and 2010 pyrosequencing reads, respectively (Table 1). Between 72.0%, 76.1% and 69.1% of these OTUs harboured single representatives, respectively, thus revealing the numerical importance of singletons in the bacterial community in the *Thymus zygis* rhizosphere. When a multiple alignment was performed, 15 clusters at a similarity of 97% grouped singletons from the pyrosequencing dataset with one or more bacterial isolates. These clusters comprised easily cultured taxa such as *Streptomyces*, *Nocardioides*, *Agreia*, *Williamsia*, *Patulibacter*, *Mycobacterium*, *Arthrobacter*, *Paenibacillus*, *Psychrobacillus* and *Pseudomonas*. Diversity based on the Shannon index was higher in the 2010 pyrosequencing dataset than in the cultured bacteria and the 16S rRNA gene clone library mainly due to the higher sampling effort offered by the second generation sequencing technology. Evenness values were also almost similar (from 0.93 to 0.97) among the three approaches (Table 1) suggesting that the community associated with the rhizosphere of *Thymus zygis* consisted of a few dominant taxa and many minority groups. This result was in agreement with the large number of singletons detected in the datasets. Rarefaction curves obtained from the sequences of the pyrosequencing dataset showed that a greater sampling effort would still be required to cover the diversity in this rhizosphere soil sample at the level of species (97% cut-off) and genus (95% cut-off) (S2A–S2D Fig). However, taking into account the recently re-evaluated thresholds by Yarza and colleagues [29] to delimit higher taxonomic ranges, the sampling effort achieved full coverage at the levels of family (90% cut-off) and class (85% cut-off). In order to evaluate the library coverage (hereafter LC) of the clone library and cultured bacteria datasets, the ratio of the actual number of OTUs observed with the Chao1 estimate of species richness (%) was calculated. According to the LC statistic, when the sampling effort is weighted, both approaches allow access at the species level with comparable diversity as observed with pyrosequencing technology (Table 1). In order to determine to what extent the functional profiles associated with the results obtained by each approach may differ, the open source R package Tax4Fun [27] was used. The results reveal that despite differences at the taxonomic level, the functional profiles for each approach are similar to each other (S4 Table).

**Table 1:** Diversity, equitability and richness indices, relative number of singletons and library coverage of OTUs defined at 3% sequence divergence.

Dataset	NS	Observed OTUs	Relative richness^a^ (%)	H´ (varH)	E	Chao1^b^	LC (%)^c^	Singletons^d^ (%)
Cultured bacteria	148	89	60.1	4.23 (0.006)	0.94	239.2 (162.5; 395.9)	37.2	72.0
Clone library	184	138	75.0	4.80 (0.003)	0.97	333.0 (251.9; 471.7)	41.4	76.1
Pyrosequencing 2010	17948	8441	47.0	8.40 (0.0001)	0.93	20352.9 (19477.4; 21297.9)	41.5	69.1
Pyrosequencing 2011	19100.3^e^	6468.0	35.9	8.11^e^ (0.0001^e^)	0.93^e^	11743.6 (11285.6; 12246.1)	54.8^e^	58.2^e^

Abbreviations: E, Shannon Wiener equitability index; H´, Shannon-Wiener index; LC, library coverage; NS, number of sequences for each dataset; OTUs, operational taxonomic units; varH', variance of H´.

^a^ Relative richness, defined as the number of OTUs observed regarding to NS

^b^ Values in brackets are lower limit and upper limit Chao1 estimates at 95% confidence interval.

^c^ LC, defined as OTUs observed/ Chao1 estimate of OTUs richness

^d^ Relative number of singletons regarding to the number of OTUs

^e^ Standard deviation lower than 5% of the average value (n = 3)

### Comparison between pyrosequencing replicates

**Fig 3:**
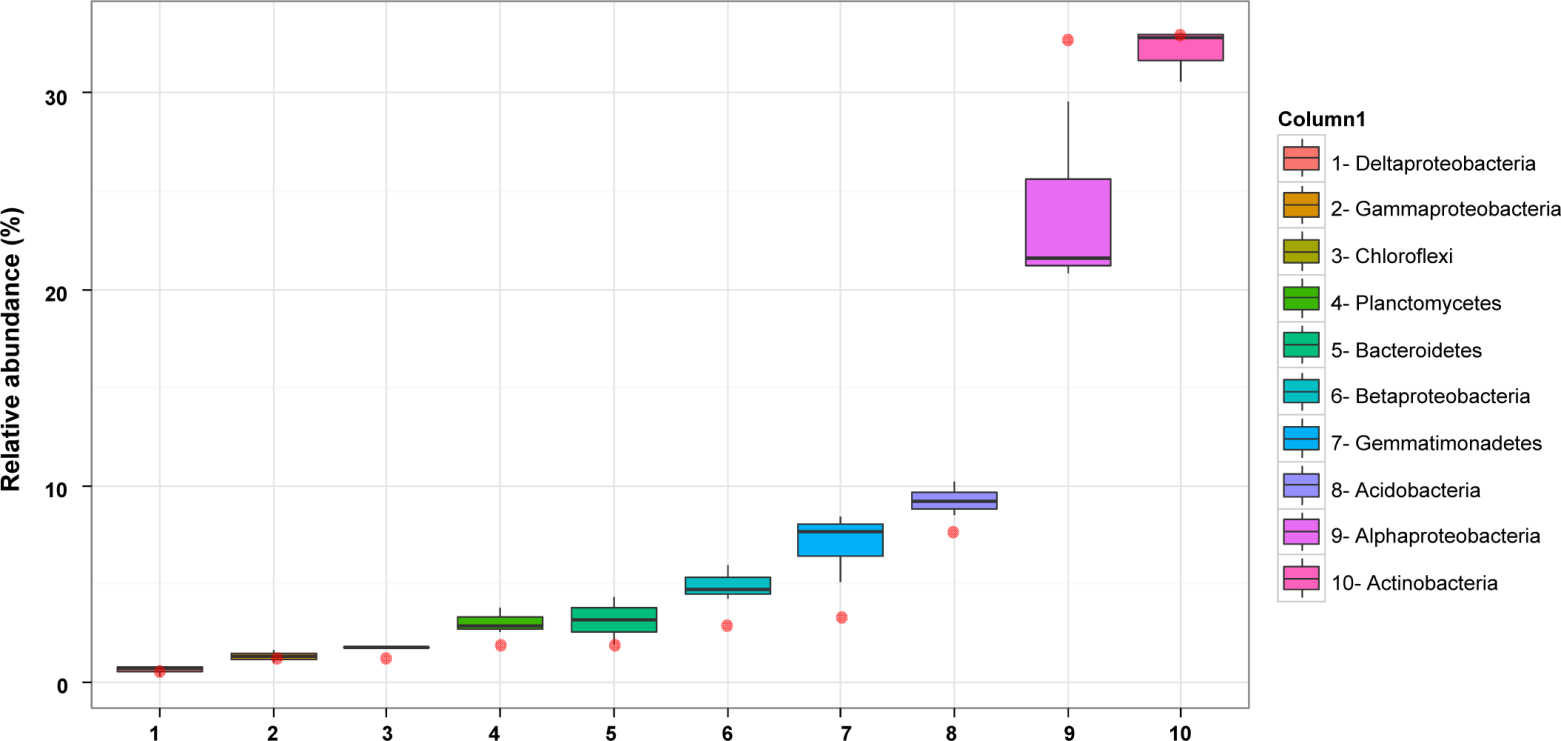
Relative abundance of the 10 most abundant phyla/ proteobacterial classes in the pyrosequencing datasets. The sample from 2010 is represented as a red point whereas three replicates from 2011 are represented as box-plots. The boxes represent the interquartile range (IQR) between the first and third quartiles (25th and 75th percentiles, respectively) and the vertical line inside the box defines the median. Whiskers represent the lowest and highest values within 1.5 times the IQR from the first and third quartiles, respectively.

To obtain a better understanding of the bacterial communities present in the rhizosphere of *Thymus zygis*, additional 454 amplicon sequences were obtained using the same 16S rRNA gene region as for the 2010 sample but instead of using metagenomic DNA from a pooled rhizosphere sample, the metagenomic DNA from the rhizosphere of three different plants sampled in 2011 were analysed separately. This resulted in a mean number of 19,100 high quality non-chimeric sequences which corresponded to a mean number of 9,175 sequences after normalization for copy number. In general, the taxonomic structures of the bacterial communities observed in the rhizosphere of the three plants collected in 2011 were similar to each other (Fig 3). The mean relative abundance (Fig 1) revealed that *Actinobacteria* (32.1% of all pyrotags), is the most represented phyla followed by *Proteobacteria* (31.6%), *Acidobacteria* (9.3%), *Gemmatimonadetes* (7.0%), *Bacteroidetes* (3.1%), *Planctomycetes* (3.1%), *Chloroflexi* (1.8%), and “*candidatu*s Saccharibacteria” (1.4%). Representatives of the candidate division WPS-1, *Armatimonadetes*, candidate division WPS-2, *Verrucomicrobia*, and *Nitrospirae*, and *Firmicutes*, were detected below 1% each, and *Parcubacteria*, candidate division BRC1, candidate division *Hydrogenedentes*, *Deinoccocus-Thermus*, *Cyanobacteria* (non-chloroplast) candidate division *Latescibacteria*, and *Fusobacteria*, below 0.1%. Several of the latter are often represented by extremely few sequences (from 1 to 6 sequences) and not always shared between all three replicate samples. Of the two most abundant phyla, within *Actinobacteria*, the subclass *Actinobacteridae* was the most numerous (21.3% of all pyrotags), followed by *Rubrobacteridae* (5.8%) and *Acidimicrobidae* (3.1%). On the other hand, among the proteobacterias, the *Alphaproteobacteria* was the largest class (23.9%), followed by *Betaproteobacteria*, *Gammaproteobacteria* and *Deltaproteobacteria* which accounted for 4.9%, 1.3% and 0.6%, respectively, of the sequences. A group of unclassified *Proteobacteria* was also detected (0.8%).

**Fig 4:**
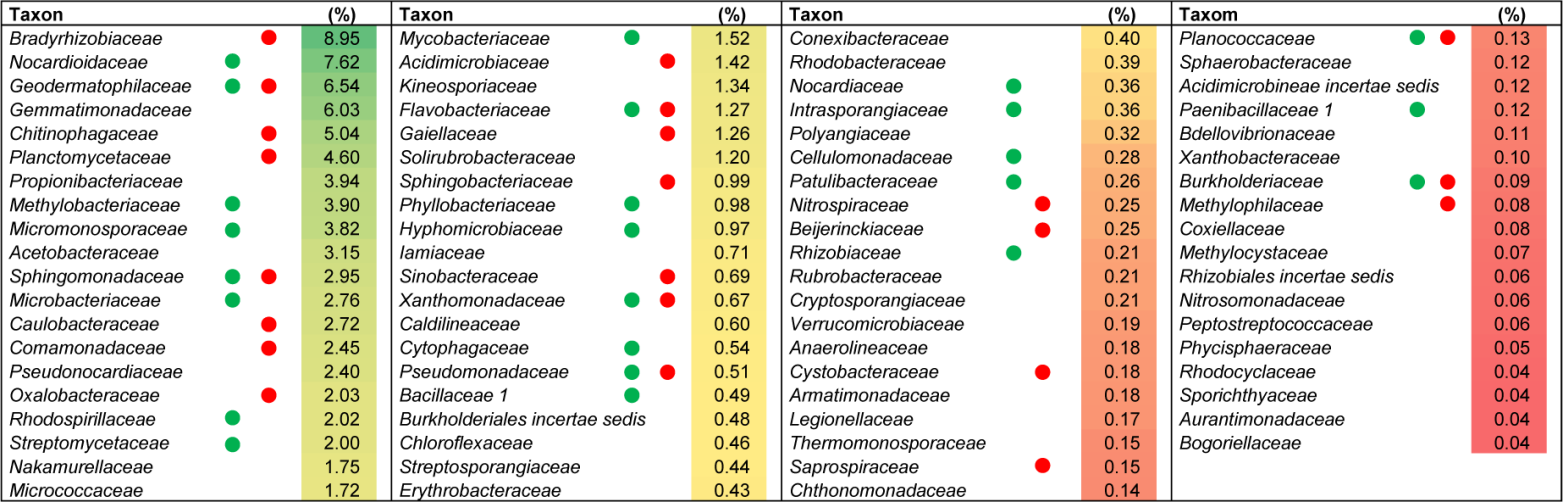
Core microbiome of the wild thymus rhizosphere at the family level. They represent bacterial families shared by all the pyrosequencing datasets (2010, 2011_1, 2011_2 and 2011_3). The heat map shows the average value (n = 4) of their relative abundances. Green or red circles indicate coincidence with families detected in cultured bacteria or the clone library, respectively.

In general, the relative abundances shifted with respect to the 2010 dataset with the largest differences observed in *Proteobacteria*, *Gemmatimonadetes*, *Acidobacteria*, *Planctomyces* and *Bacteriodetes* (Fig 1, Fig 3). Moreover, ultra-low-represented phyla appeared in some of the replicate samples from 2011 but not in the 2010 sample (Fig 2: asterisks) thereby increasing the total number of different phyla/ candidate divisions detected, but this had a smaller relative effect on the total number of different classes, orders, families and genera (S1 Fig). According to the diversity indices (Table 1), the replicate pyrosequencing samples of 2011 show lower diversity and relative richness than the pyrosequencing dataset from 2010 but also a proportionally lower percentage of singleton reads, as could be expected taking into account the higher sampling effort. Nevertheless, and similar to the 2010 sample, between 11 to 13 of the singletons from each replicate dataset clustered at a similarity of 97% with one or more bacterial isolates. According to the principal coordinates analysis (PCoA) plot based on weighted UniFrac distances, the three replicates of 2011 and that of 2010 (pooled sample) were placed more or less equidistant from each other, as explained by 63.2%, 26.1% and 10.7% of the observed differences at variable 1, 2 and 3 respectively (S3 Fig). This indicates that the bacterial community showed, at least seasonally, the same taxonomic pattern during two consecutive years and hence it might be a stabilized community. The similarity between all the pyrosequenced amplicon libraries was also revealed by the number of families shared. This permitting the definition of the core microbiome of the rhizosphere of the *Thymus zygis* plant (Fig 4) which is constituted of a total of 78 different families (accounting for a relative abundance ≥ 0.14% of the total community). The most highly represented families (more than 3%) found in this core microbiome were *Bradyrhizobiaceae*, *Nocardioidaceae*, and *Geodermatophilaceae* followed by other families belonging to *Actinobacteria* or *Alphaproteobacteria* as well as *Gemmatimonadetes*, *Bacteroidetes* and *Planctomycetes*. Most of the families which make up the core microbiome were also found in the clone library and/or cultured-dependent approach (Fig 4), confirming qualitatively the prevalence of these families independently of the methodology used to study the rhizospheric bacterial community. The functional profile of the core microbiome of the rhizososphere of *Thymus zygis* inferred with Tax4Fun (S4 Table) suggested that the overall functional structure of the community was dominated by KEGG pathways related to metabolism especially that of carbohydrates (starch, sucrose, amino sugars and nucleotide sugars) and nitrogen-containing compounds such as amino acids and nucleotides (arginine, proline, glycine, serine, threonine, purine and pyrimidine among others). With regard to energy metabolism, genes related with the nitrogen metabolism, oxidative phosphorylation, methane metabolism and carbon fixation pathways in prokaryotes dominated. The metabolism of terpenoids, polyketides, lipids, xenobiotics, and glycans were also predicted but at lower abundances. Another dominant KEGG category of the inferred functional profile of the core rhizosphere bacterial community is associated with environmental information processing, principally in pathways related to membrane transport by ABC transporters and signal transduction by two component systems.

## Discussion

In this work, the diversity within the rhizosphere bacterial community associated with a wild thyme species from the Sierra Nevada National Park was explored using culture-dependent and independent approaches. The clone library and multiplex amplicon pyrosequencing permit access to the prokaryotic diversity at high resolutions, including low-abundance species of the so-called “rare biosphere” [30,31] and/or bacteria resistant to *in vitro* culturing strategies belonging to uncultured “dark matter” clades [32,33]. On the other hand, the cultured bacteria complement the molecular approaches and will permit further in-depth metabolic, physiological and genomic characterization and thereby the possibility to obtain a better understanding of their roles in the rhizosphere of *Thymus zygis* plants. This work is currently in progress.

In this study, the percentage of isolates that could be cultivated *in vitro* (cultivability) compared to the total viable bacteria present in the sample was 0.60% which is within the range of values (0.01% to 1%) obtained in other solid media based studies [34,35]. Although relatively low diversity was recovered compared to the molecular techniques used in this study, the relative abundance of members of the four phyla detected appears to be proportionally more similar to those observed in the pyrosequencing datasets than to the clone library. This suggests that within the constraints faced for effective cultivation, the media and conditions used in this study to culture microorganisms *in vitro* successfully recovered a representative subset of the true diversity present, including numerically non-dominant taxa. Moreover, in spite of the difficulty to isolate “uncultured bacteria” a strain belonging to the subclass *Rubrobacteridae* was successfully isolated [36]. Although this taxon was also detected by both molecular techniques and is ubiquitous in the soil environment, only a few known cultivable representatives of this subclass have been identified [13,36]. In addition to this isolate, 4.7% of the isolates identified in this study shared a 16S rRNA gene similarity of less than 97% with their closest type strains, and thus may constitute new bacterial taxa [37]. In fact, if the sequence identity threshold of 98.65% suggested by Kim *et al*. [38] is used to differentiate species, 37.8% of all the strains isolated from the rhizosphere soil sample in this study may represent new bacterial species.

The molecular techniques used to view the full bacterial diversity associated with the rhizosphere of *Thymus zygis* consisted of a clone library of near full length 16S rRNA gene sequences but with low depth and amplicon pyrosequencing of short 16S rRNA gene fragments at very high depth. Surprisingly, members of several dominant phyla which had been successfully cultured were not recovered in the clone library and the relative abundances of dominant taxa were not in agreement with those observed with the pyrosequencing technique. According to the RDP probe match tool [20], *a priori*, coverage of the primers used to amplify 16S rRNA genes from metagenomic DNA does not account for these differences. However, during the clone library construction other factors such as the soil metagenomic DNA quality, PCR, cloning biases during ligation and transformation steps, etc. may affect the outcome and thereby distort the perception of the bacterial community structure. As a result other authors have also observed that 16S rRNA gene clone libraries may not represent a complete or accurate picture of the true bacterial community [39,40]. Another unexpected result was the detection of representatives of the lineage *Ignavibacteriae* in the clone library but not in the pyrosequencing dataset regardless of the much higher sampling effort of the latter. As pointed out above, when sequences identified as *Ignavibacteriae* were trimmed to a fragments resembling both in size and gene regions the 16S rRNA pyrotags, it could no longer be identified. This result highlights the limitation of taxonomic assignment methods and current databases for the correct identification of short fragments of the 16S rRNA gene.

Although the large differences in the sampling effort between the approaches used do not permit accurate comparative studies, the clone library and isolates may complement qualitatively the pyrosequencing dataset by corroborating the presence of certain taxa and to permit a more accurate taxonomic assignment of those taxa. In this manner, the cultured isolates can also be used to benchmark data obtained by the molecular approaches by permitting the verification of singleton sequences. We have observed that 11 to 15 of the singletons detected in each pyrosequencing dataset clustered with isolated bacteria. Therefore, a proportion of the singleton sequences of the pyrosequencing dataset correspond to real but underrepresented bacteria and consequently suggest that conservative strategies that eliminate singletons as sequencing artefacts should take into account this possible loss of information especially when the “rare biosphere” is being sought. This fact also confirms that the cultured approach permits access to the “rare biosphere” [41].

The overall diversity observed in the rhizosphere of *Thymus zygis* reveals an abundance of *Proteobacteria* (predominantly *Alphaproteobacteria* and *Betaproteobacteria* classes), *Actinobacteria*, *Acidobacteria*, *Gemmatimonadetes*, *Bacteroidetes*, *Planctomycetes*, *Chloroflexi*, and “*candidatus* Saccharibacteria”. Although the order of predominance may vary, all of the mentioned phyla together with *Firmicutes* are usually abundant in soil in general [39,42] and the rhizosphere in particular such as those associated with a number of different plants such as oak [43], aspen [44], potato [45], *Arabidopsis* spp. [4,46,47], cactus [48], cucumber [49], artic grasses [50], maize [51,52], Japanese barberry [53], cannabis [54], medicinal plants [55,56], rice [57], soybean [58], wheat [59,60] and creosote plants [61], amongst others. Aside from effects due to edaphic characteristics such as soil pH [62], the variations in phyla predominance observed in the rhizosphere bacterial communities associated with the different plants could depend on the nutrients released by the plant in the rhizosphere micro-niche where decomposition of plant-derived carbon sources favour faster growing *Proteobacteria* and *Bacteriodetes* copiotrophs while diminishing slower growing oligotrophs such as *Acidobacteria* [63]. More recently, it was shown that wheat root derived carbon was dominantly assimilated by *Proteobacteria* and *Actinobacteria* [60]. The fact that many strains belonging to *Actinobacteria* have been described, on the one hand, as plant-growth promoting bacteria and as producers of a wide range of biologically active secondary metabolites [64], and on the other to have reputedly high resistance traits to desiccation and starvation may explain their enormous versatility and ubiquitous presence in the rhizosphere and in soil in general.

Deeper analysis of the rhizosphere bacterial communities of *Thymus zygis* using amplicon pyrosequencing permitted the definition of a core microbiome for this niche. The most abundant families observed within this core microbiome belong to the alphaproteobacterial orders *Rhizobiales*, *Rhodospirilalles*, *Sphingomonadales* and *Caulobacteriales*. Many of these appear to dominantly assimilate wheat root derived carbon together with the actinobacterial orders *Micrococcales*, *Acidimicrobiales*, and *Propionibacteriales* [60]. Besides the generally heterotrophic nature of the abundant families found in the core microbiome, the presence of *Methylobacteriaceae* and *Hyphomicrobiaceae* which both have methylotrophic members suggests an importance of single carbon compounds such as methane or methanol as C sources in the rhizosphere. On the other hand, the actinobacterial families *Micromonosporaceae* and *Pseudonocacardiaceae* have been associated with senescing wheat roots [59] and in general *Actinobacteria* have been reported to have increased numbers on older roots [65]. Within the alphaproteobacterial and actinobacterial families, abundant genera are found which include important xenobiotic degraders such as *Sphingomonas*, *Phenylobacterium*, and *Patulibacter*. Therefore, it appears that at least part of the core microbiome associated with the *Thymus zygis* rhizosphere may have been selected by the plant in reponse to rhizodeposition composition while others may be related to the degradation of complex molecules associated with older scenescing roots or plant derived secondary products. On the other hand, the abundance of the family *Bradyrhizobiaceae*, which include *Bradyrhizobium* and other genera which participate in the nitrogen cycle [66] could indicate an important role for this cycle in this niche. This is also supported by the presence of possible nitrifying genera associated with *Nitrospiraceae* and that many of the taxa identified in our datasets coincide with those associated with nitrogen cycle genes in the rhizosphere of holm oak [67] growing within the same geographical region. Altogether this suggests that the main drivers of the bacterial community in the *Thymus zygis* rhizosphere might be related with the plant-bacteria interchange of nutrients and their participation in the biogeochemical cycles.

To obtain a better understanding of the possible gene functions associated with the taxa in the core microbiome, the Tax4Fun prediction tool was used. The major functional category that could be inferred in the rhizosphere of *Thymus zygis* was metabolism especially that of carbohydrates and amino acids which are typical components of root exudates. However, also secondary metabolite degradation functions could be detected including those of geraniol, limonene and pinene which have been associated with *Thymus zygis* [68]. The abundance of functional groups related to the biodegradation and metabolism of xenobiotics such as benzoates, aminobenzoate and bisphenol, may also be related to the presence of complex secondary metabolites or polymers with aromatic structures which may be released by the plants into the rhizosphere or form part of lignin in woody roots. A high abundance of functions related to energy metabolism, including nitrogen metabolism, and the abundance of transporters and two component systems imply an exchange of nutrients and signals. Therefore, the functional profile inferred by the prediction tool are similar to those which may be attributed to the more abundant families of the core microbiome especially with regard to the importance of metabolism of simple and complex carbon and nitrogen sources which may include methane, xenobiotics, and secondary metabolites such as terpenoids or complex polymers. This functional profile suggests that the bacterial community shares an intricate relationship with the roots of this aromatic plant, presumably allowing a feedback ecological benefit.

In conclusion, by using three different but complementary approaches to explore, for the first time, the rhizosphere bacterial community of this medicinal and culinary important plant has revealed key bacterial families which may have undergone selection by the plant. Further investigation facilitated by the availability of a collection of cultured bacteria is currently underway in order to elucidate how the identified bacterial families function and respond to environmental changes and benefit wild thyme plant growth.

## Supporting Information

S1 Fig Number of taxa recovered at the different taxonomic levels, from phylum to genus, with each of the three approaches used in this study. Asterisks indicate a standard deviation inferior to 8% for the 2011 pyrosequencing datasets (n = 3).
(TIF)

S2 Fig Rarefaction curves of 16S rRNA gene sequences from the 454 pyrosequencing datasets calculated with A) 3%, B) 5%, C) 10% and D) 15% distance cut-offs. (TIF)

S3 Fig Principal Coordinate Analysis (PCoA) based on weighted Unifrac distances of bacterial community inhabiting the rhizosphere soil of wild thyme based on the pyrosequencing dataset from 2010 (blue) and the three replicates from 2011 (1–3, red). (TIF)

S1 Table Soil physicochemical properties. (DOCX)

S2 Table Taxonomic diversity of cultured bacteria based on their 16S rRNA gene sequences. (DOCX)

S3 Table Taxonomic diversity of clone library based on their 16S rRNA gene sequences. (DOCX)

S4 Table Percentages (>0.5%) of 1, 2 and 3 tier KEGG Orthology (KO) categories predicted from each 16S rRNA dataset with the Tax4Fun tool. 1, Cultured bacteria; 2, Clone library; 3, Pyrosequencing 2010; 4, Pyrosequencing 2010; 5, Core microbiome.
(DOCX)
