# Supplementary figures and images for: Assessing Bacterial Diversity in the Rhizosphere of Thymus zygis Growing in the Sierra Nevada National Park (Spain) through Culture-Dependent and Independent Approaches
Source: PLoS One. 2016 Jan 7;11(1):e0146558. doi: 10.1371/journal.pone.0146558 (PMC4711807; doi:10.1371/journal.pone.0146558)

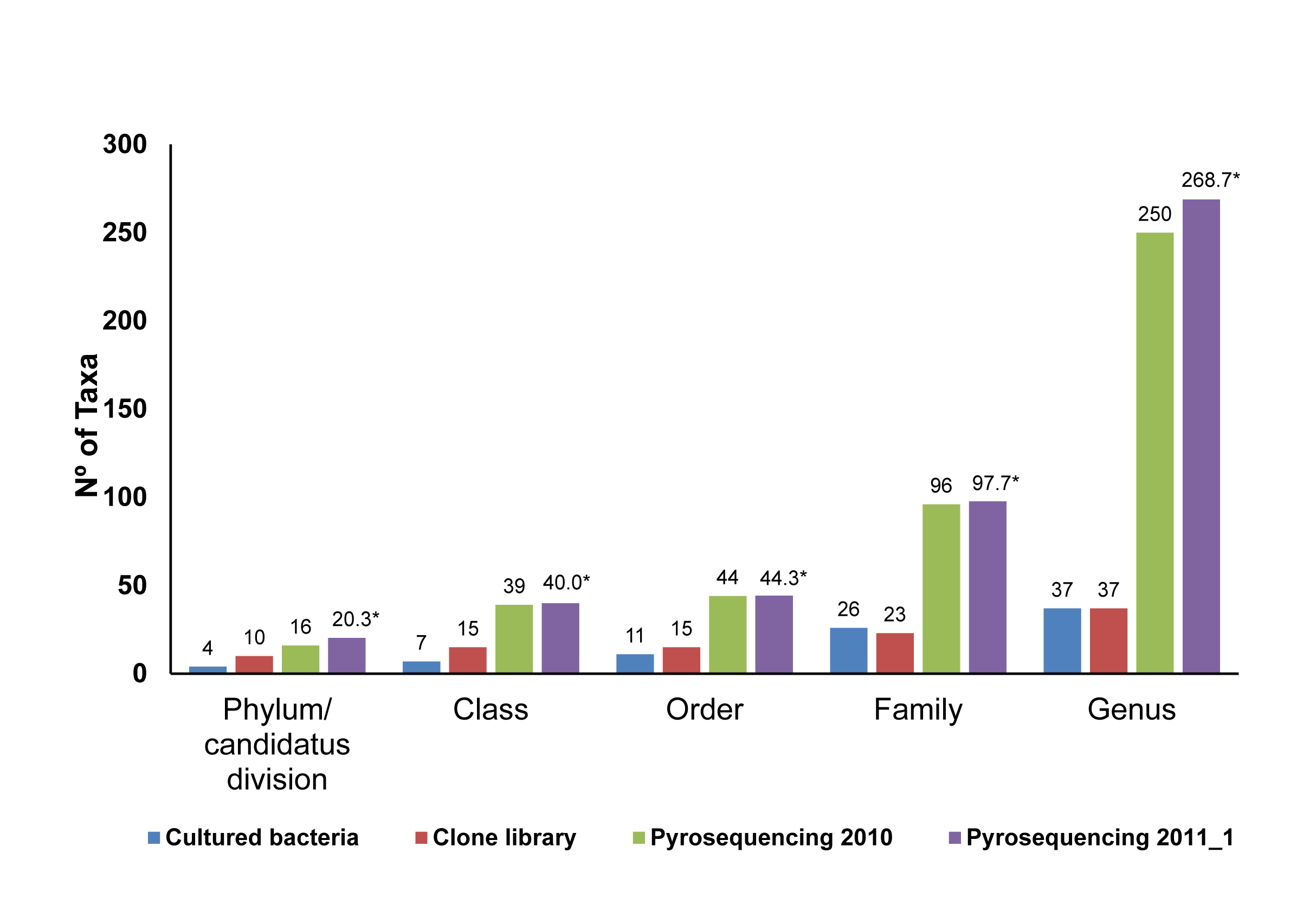

Supplement: S1 Fig — Asterisks indicate a standard deviation inferior to 8% for the 2011 pyrosequencing datasets (n = 3). (TIF) [file pone.0146558.s001.tif]

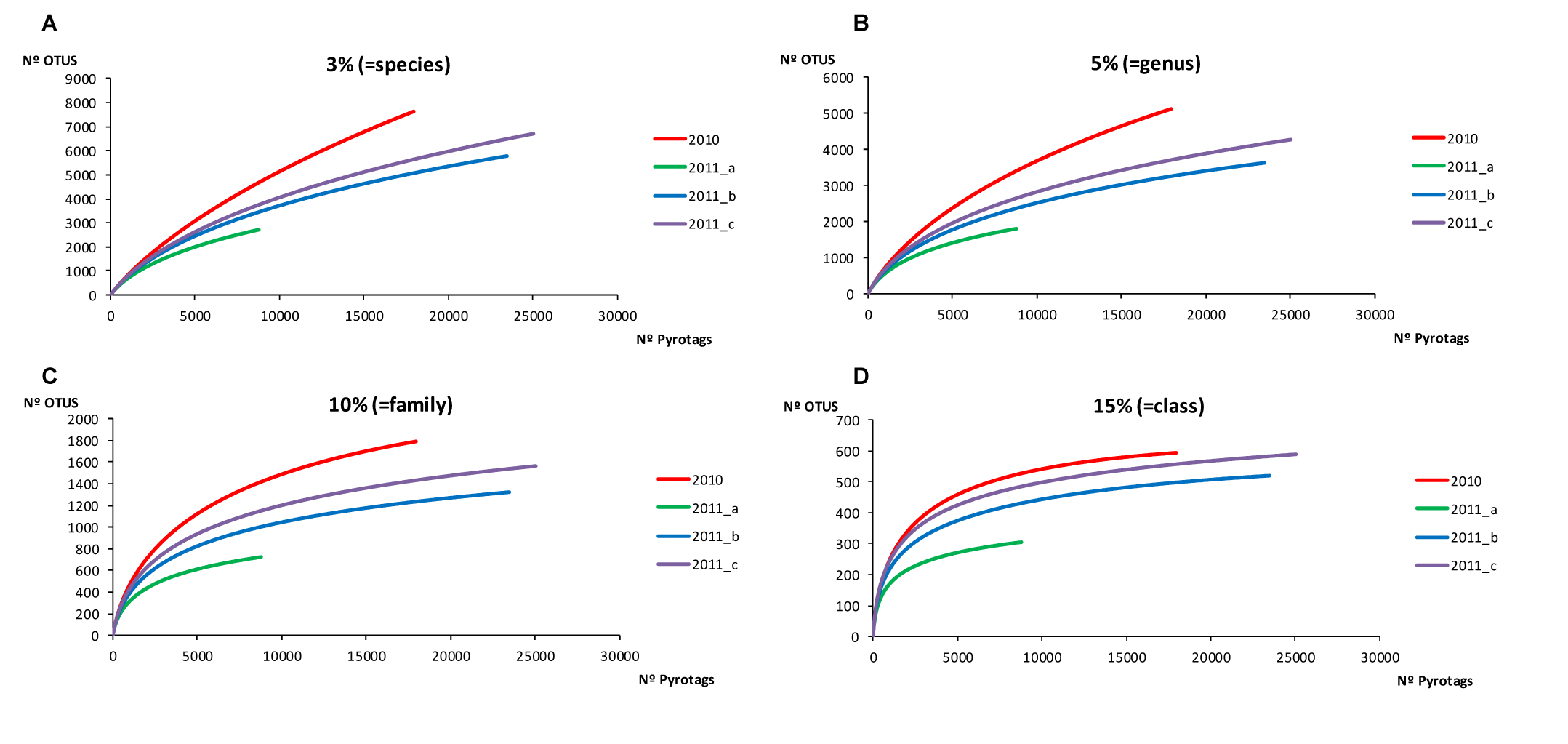

Supplement: S2 Fig — (TIF) [file pone.0146558.s002.tif]

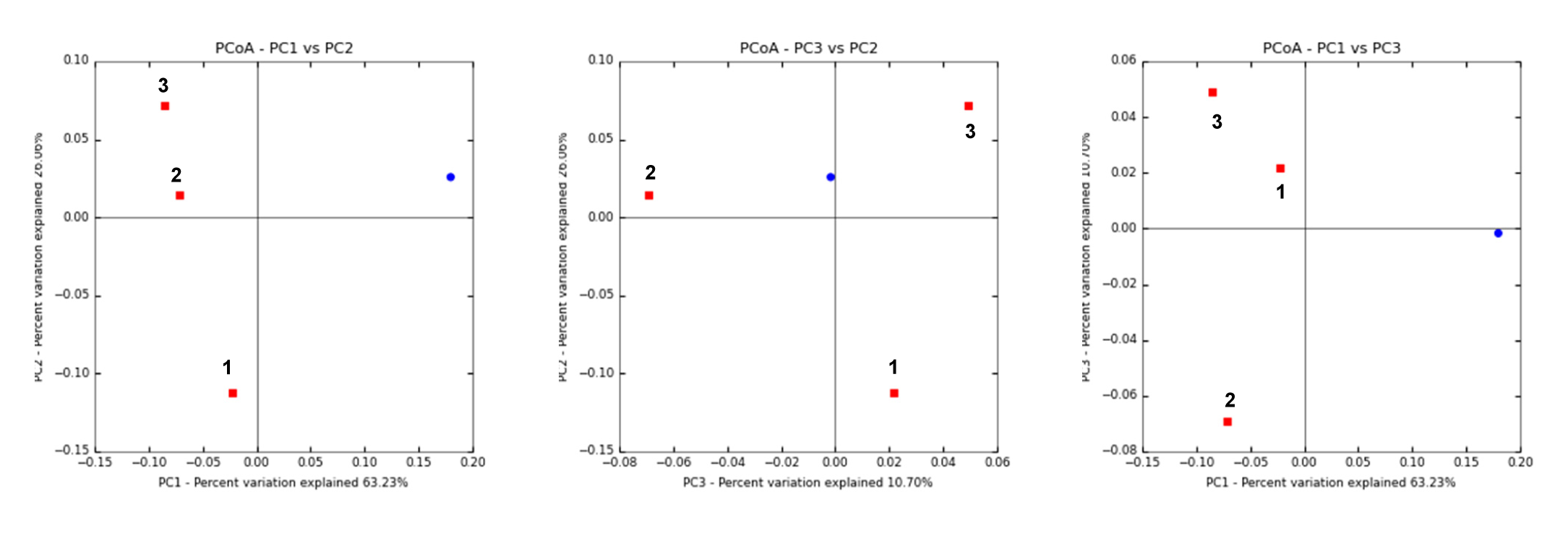

Supplement: S3 Fig — (TIF) [file pone.0146558.s003.tif]
