# Supplementary material for: Assessing Bacterial Diversity in the Rhizosphere of Thymus zygis Growing in the Sierra Nevada National Park (Spain) through Culture-Dependent and Independent Approaches
Source: PLoS One. 2016 Jan 7;11(1):e0146558. doi: 10.1371/journal.pone.0146558 (PMC4711807; doi:10.1371/journal.pone.0146558)
Supplement: S1 Table — (DOCX) [file pone.0146558.s004.docx]

**Table S1.** Soil physicochemical properties.

| **Test description** |  |
| --- | --- |
| Active lime | Not detected % |
| Carbonates | 1.6% |
| Classification Type | sandy loam |
| Assimilable phosphorus | 3 ppm |
| Humic matter | 1.24% |
| Total nitrogen | 0.082% |
| pH | 6.8 |
| Assimilable potassium | 130 ppm |
| Salinity pretest | 0.04 mmhos cm^-1^ |
| Clay texture | 8.0% |
| Sand texture | 68.68% |
| Silt texture | 23.32% |
