# Supplementary material for: Assessing Bacterial Diversity in the Rhizosphere of Thymus zygis Growing in the Sierra Nevada National Park (Spain) through Culture-Dependent and Independent Approaches
Source: PLoS One. 2016 Jan 7;11(1):e0146558. doi: 10.1371/journal.pone.0146558 (PMC4711807; doi:10.1371/journal.pone.0146558)
Supplement: S2 Table — (DOCX) [file pone.0146558.s005.docx]

**Table S2.** Taxonomic diversity of cultured bacteria based on their 16S rRNA gene sequences

| **Strain** | **Accession number** | **Phylum (Class)^a^** | **Closest type strain^c^** | **Similarity (%)** |
| --- | --- | --- | --- | --- |
| CB-281479 | JX841017 | *Actinobacteria* (*Actinobacteridae*) | *Aeromicrobium ginsengisoli* (AB245394) | 99.3 |
| CB-281429 | JX840988 | *Actinobacteria* (*Actinobacteridae*) | *Agreia pratensis* (AJ310412) | 99.8 |
| CB-281539 | JX841074 | *Actinobacteria* (*Actinobacteridae*) | *Agrococcus jenensis* (X92492) | 99.5 |
| CB-281406 | JX840965 | *Actinobacteria* (*Actinobacteridae*) | *Arthrobacter globiformis* (X80736) | 99.8 |
| CB-281417 | JX840976 | *Actinobacteria* (*Actinobacteridae*) | *Arthrobacter oryzae* (AB279889) | 98.4 |
| CB-281426 | JX840985 | *Actinobacteria* (*Actinobacteridae*) | *Arthrobacter oryzae* (AB279889) | 99.0 |
| CB-281518 | JX841056 | *Actinobacteria* (*Actinobacteridae*) | *Arthrobacter oxydans* (X83408) | 98.3 |
| CB-281411 | JX840970 | *Actinobacteria* (*Actinobacteridae*) | *Arthrobacter oxydans* (X83408) | 99.2 |
| CB-281409 | JX840968 | *Actinobacteria* (*Actinobacteridae*) | *Arthrobacter oxydans* (X83408) | 99.4 |
| CB-281427 | JX840986 | *Actinobacteria* (*Actinobacteridae*) | *Arthrobacter oxydans* (X83408) | 99.5 |
| CB-281410 | JX840969 | *Actinobacteria* (*Actinobacteridae*) | *Arthrobacter phenanthrenivorans* (AM176541) | 98.5 |
| CB-281408 | JX840967 | *Actinobacteria* (*Actinobacteridae*) | *Arthrobacter phenanthrenivorans* (AM176541) | 98.8 |
| CB-281416 | JX840975 | *Actinobacteria* (*Actinobacteridae*) | *Arthrobacter phenanthrenivorans* (AM176541) | 99.3 |
| CB-281407 | JX840966 | *Actinobacteria* (*Actinobacteridae*) | *Arthrobacter ramosus* (X80742) | 100 |
| CB-281414 | JX840973 | *Actinobacteria* (*Actinobacteridae*) | *Arthrobacter ramosus* (X80742) | 99.6 |
| CB-281415 | JX840974 | *Actinobacteria* (*Actinobacteridae*) | *Arthrobacter ramosus* (X80742) | 99.6 |
| CB-281413 | JX840972 | *Actinobacteria* (*Actinobacteridae*) | *Arthrobacter ramosus* (X80742) | 99.7 |
| CB-281412 | JX840971 | *Actinobacteria* (*Actinobacteridae*) | *Arthrobacter ramosus* (X80742) | 99.8 |
| CB-281418 | JX840977 | *Actinobacteria* (*Actinobacteridae*) | *Arthrobacter sulfonivorans* (AF235091) | 99.4 |
| CB-281465 | JX841003 | *Actinobacteria* (*Actinobacteridae*) | *Blastococcus saxobsidens* (FN600641) | 98.5 |
| CB-281439 | JX840996 | *Actinobacteria* (*Actinobacteridae*) | *Cellulomonas aerilata* (EU560979) | 100 |
| CB-281440 | JX840997 | *Actinobacteria* (*Actinobacteridae*) | *Cellulomonas cellasea* (X83804) | 99.9 |
| CB-281425 | JX840984 | *Actinobacteria* (*Actinobacteridae*) | *Cellulomonas chitinilytica* (AB268586) | 98.1 |
| CA-281456 | JX840953 | *Actinobacteria* (*Actinobacteridae*) | *Kitasatospora kifunensis* (U93322) | 99.6 |
| CB-281522 | JX841058 | *Actinobacteria* (*Actinobacteridae*) | *Kribbella ginsengisoli* (AB245391) | 100 |
| CB-281431 | JX840989 | *Actinobacteria* (*Actinobacteridae*) | *Leifsonia kafniensis* (AM889135) | 98.4 |
| CB-281419 | JX840978 | *Actinobacteria* (*Actinobacteridae*) | *Microbacterium flavescens* (Y17232) | 98.6 |
| CB-281432 | JX840990 | *Actinobacteria* (*Actinobacteridae*) | *Microbacterium phyllosphaerae* (AJ277840) | 99.0 |
| CB-281524 | JX841059 | *Actinobacteria* (*Actinobacteridae*) | *Microbacterium terricola* (AB234025) | 98.1 |
| CB-281519 | JX841057 | *Actinobacteria* (*Actinobacteridae*) | *Micromonospora lupine* (AJ783996) | 99.7 |
| CB-281466 | JX841004 | *Actinobacteria* (*Actinobacteridae*) | *Modestobacter versicolor* (AJ871304) | 99.6 |
| CB-281487 | JX841025 | *Actinobacteria* (*Actinobacteridae*) | *Mycobacterium austroafricanum* (X93182) | 97.3 |
| CB-281540 | JX841075 | *Actinobacteria* (*Actinobacteridae*) | *Mycobacterium austroafricanum* (X93182) | 97.5 |
| CB-281525 | JX841060 | *Actinobacteria* (*Actinobacteridae*) | *Mycobacterium austroafricanum* (X93182) | 98.6 |
| CB-281541 | JX841076 | *Actinobacteria* (*Actinobacteridae*) | *Mycobacterium austroafricanum* (X93182) | 98.8 |
| CB-281480 | JX841018 | *Actinobacteria* (*Actinobacteridae*) | *Mycobacterium canariasense* (AY255478) | 98.6 |
| CB-281485 | JX841023 | *Actinobacteria* (*Actinobacteridae*) | *Mycobacterium hodleri* (X93184) | 98.5 |
| CB-281486 | JX841024 | *Actinobacteria* (*Actinobacteridae*) | *Mycobacterium hodleri* (X93184) | 98.8 |
| CB-281492 | JX841030 | *Actinobacteria* (*Actinobacteridae*) | *Nocardia ninae* (DQ235687) | 98.6 |
| CB-281491 | JX841029 | *Actinobacteria* (*Actinobacteridae*) | *Nocardia salmonicida* (AF430050) | 97.3 |
| CB-281543 | JX841078 | *Actinobacteria* (*Actinobacteridae*) | *Nocardioides caricicola* (FJ750845) | 100 |
| CB-281545 | JX841080 | *Actinobacteria* (*Actinobacteridae*) | *Nocardioides caricicola* (FJ750845) | 98.0 |
| CB-281542 | JX841077 | *Actinobacteria* (*Actinobacteridae*) | *Nocardioides caricicola* (FJ750845) | 98.2 |
| CB-281544 | JX841079 | *Actinobacteria* (*Actinobacteridae*) | *Nocardioides caricicola* (FJ750845) | 98.4 |
| CB-281476 | JX841014 | *Actinobacteria* (*Actinobacteridae*) | *Nocardioides exalbidus* (AB273624) | 96.2 |
| CB-281526 | JX841061 | *Actinobacteria* (*Actinobacteridae*) | *Nocardioides exalbidus* (AB273624) | 97.8 |
| CB-281474 | JX841012 | *Actinobacteria* (*Actinobacteridae*) | *Nocardioides exalbidus* (AB273624) | 98.7 |
| CB-281473 | JX841011 | *Actinobacteria* (*Actinobacteridae*) | *Nocardioides furvisabuli* (DQ411542) | 98.5 |
| CB-281527 | JX841062 | *Actinobacteria* (*Actinobacteridae*) | *Nocardioides hankookensis* (EF555584) | 97.1 |
| CB-281546 | JX841081 | *Actinobacteria* (*Actinobacteridae*) | *Nocardioides hankookensis* (EF555584) | 97.3 |
| CB-281489 | JX841027 | *Actinobacteria* (*Actinobacteridae*) | *Nocardioides hankookensis* (EF555584) | 97.8 |
| CB-281481 | JX841019 | *Actinobacteria* (*Actinobacteridae*) | *Nocardioides hankookensis* (EF555584) | 98.1 |
| CB-281471 | JX841009 | *Actinobacteria* (*Actinobacteridae*) | *Nocardioides hankookensis* (EF555584) | 99.6 |
| CB-281478 | JX841016 | *Actinobacteria* (*Actinobacteridae*) | *Nocardioides hwasunensis* (AM295258) | 96.5 |
| CB-281475 | JX841013 | *Actinobacteria* (*Actinobacteridae*) | *Nocardioides hwasunensis* (AM295258) | 97.1 |
| CB-281477 | JX841015 | *Actinobacteria* (*Actinobacteridae*) | *Nocardioides hwasunensis* (AM295258) | 97.7 |
| CB-281490 | JX841028 | *Actinobacteria* (*Actinobacteridae*) | *Nocardioides hwasunensis* (AM295258) | 97.7 |
| CB-281547 | JX841082 | *Actinobacteria* (*Actinobacteridae*) | *Nocardioides hwasunensis* (AM295258) | 98.2 |
| CB-281467 | JX841005 | *Actinobacteria* (*Actinobacteridae*) | *Nocardioides iriomotensis* (AB544079) | 96.9 |
| CB-281548 | JX841083 | *Actinobacteria* (*Actinobacteridae*) | *Nocardioides kribbensis* (AY835924) | 97.8 |
| CB-281468 | JX841006 | *Actinobacteria* (*Actinobacteridae*) | *Nocardioides lianchengensis* (HQ657322) | 100 |
| CB-281472 | JX841010 | *Actinobacteria* (*Actinobacteridae*) | *Nocardioides pyridinolyticus* (U61298) | 97.7 |
| CB-281469 | JX841007 | *Actinobacteria* (*Actinobacteridae*) | *Phycicoccus badiiscoriae* (FN386744 ) | 97.0 |
| CB-281488 | JX841026 | *Actinobacteria* (*Actinobacteridae*) | *Rhodococcus jostii* (AB046357) | 99.2 |
| CA-281520 | JX840962 | *Actinobacteria* (*Actinobacteridae*) | *Steptomyces candidus* (DQ026663) | 99.9 |
| CA-281523 | JX840964 | *Actinobacteria* (*Actinobacteridae*) | *Steptomyces kurssanovii* (AB184325) | 98.6 |
| CA-281446 | JX840944 | *Actinobacteria* (*Actinobacteridae*) | *Streptomyces atroolivaceus* (AJ781320) | 98.7 |
| CA-281461 | JX840958 | *Actinobacteria* (*Actinobacteridae*) | *Streptomyces avidinii* (AB184395) | 99.5 |
| CA-281457 | JX840954 | *Actinobacteria* (*Actinobacteridae*) | *Streptomyces badius* (AY999783) | 99.7 |
| CA-281447 | JX840945 | *Actinobacteria* (*Actinobacteridae*) | *Streptomyces brasiliensis* (AB249981) | 98.0 |
| CA-281448 | JX840946 | *Actinobacteria* (*Actinobacteridae*) | *Streptomyces canus* (AY999775) | 98.2 |
| CA-281450 | JX840947 | *Actinobacteria* (*Actinobacteridae*) | *Streptomyces canus* (AY999775) | 98.2 |
| CA-281453 | JX840950 | *Actinobacteria* (*Actinobacteridae*) | *Streptomyces chrysomallus* (AB184120) | 99.4 |
| CA-282244 | JX841091 | *Actinobacteria* (*Actinobacteridae*) | *Streptomyces ciscaucasicus* (AB184208) | 99.9 |
| CA-281521 | JX840963 | *Actinobacteria* (*Actinobacteridae*) | *Streptomyces clavuligerus* (AY999718) | 96.9 |
| CA-281458 | JX840955 | *Actinobacteria* (*Actinobacteridae*) | *Streptomyces cyaneofuscatus* (AB184860) | 96.4 |
| CA-281459 | JX840956 | *Actinobacteria* (*Actinobacteridae*) | *Streptomyces cyaneofuscatus* (AB184860) | 99.1 |
| CA-281454 | JX840951 | *Actinobacteria* (*Actinobacteridae*) | *Streptomyces durhamensis* (AY999785) | 97.2 |
| CA-281455 | JX840952 | *Actinobacteria* (*Actinobacteridae*) | *Streptomyces eurythermus* (D63870) | 98.9 |
| CA-281460 | JX840957 | *Actinobacteria* (*Actinobacteridae*) | *Streptomyces fimicarius* (AY999784) | 99.5 |
| CA-282243 | JX841090 | *Actinobacteria* (*Actinobacteridae*) | *Streptomyces galilaeus* (AB045878) | 100 |
| CA-281451 | JX840948 | *Actinobacteria* (*Actinobacteridae*) | *Streptomyces galilaeus* (AB045878) | 98.8 |
| CA-281462 | JX840959 | *Actinobacteria* (*Actinobacteridae*) | *Streptomyces libani* (AJ781351) | 99.7 |
| CA-281452 | JX840949 | *Actinobacteria* (*Actinobacteridae*) | *Streptomyces microflavus* (DQ445795) | 99.1 |
| CA-281463 | JX840960 | *Actinobacteria* (*Actinobacteridae*) | *Streptomyces novaecaesareae* (AB184357) | 100 |
| CA-281464 | JX840961 | *Actinobacteria* (*Actinobacteridae*) | *Streptomyces tauricus* (AB045879) | 98.3 |
| CB-281484 | JX841022 | *Actinobacteria* (*Actinobacteridae*) | *Williamsia faeni* (DQ157929) | 98.4 |
| CB-281483 | JX841021 | *Actinobacteria* (*Actinobacteridae*) | *Williamsia faeni* (DQ157929) | 98.9 |
| CB-281482 | JX841020 | *Actinobacteria* (*Actinobacteridae*) | *Williamsia limnetica* (HQ157192) | 98.8 |
| CB-281470 | JX841008 | *Actinobacteria* (*Actinobacteridae*) | *Nocardioides caricicolan* (FJ750845) | 98.0 |
| CB-281549 | JX841084 | *Actinobacteria* (*Rubrobacteridae*) | *Patulibacter minatonensis* (AB193261) | 97.8 |
| CB-281445 | JX841002 | *Bacteroidetes* (*Flavobacteria*) | *Flavobacterium frigidimaris* (AB183888) | 99.6 |
| CB-281534 | JX841069 | *Bacteroidetes* (*Sphingobacteria*) | *Hymenobacter metalli* (HM032898) | 97.3 |
| CB-281434 | JX840991 | *Firmicutes* (*Bacilli*) | *Bacillus muralis* (AJ316309) | 99.9 |
| CB-281435 | JX840992 | *Firmicutes* (*Bacilli*) | *Bacillus simplex* (AJ439078) | 99.6 |
| CB-281436 | JX840993 | *Firmicutes* (*Bacilli*) | *Bacillus simplex* (AJ439078) | 99.6 |
| CB-281535 | JX841070 | *Firmicutes* (*Bacilli*) | *Bacillus simplex* (AJ439078) | 99.6 |
| CB-281437 | JX840994 | *Firmicutes* (*Bacilli*) | *Bacillus simplex* (AJ439078) | 99.7 |
| CB-281420 | JX840979 | *Firmicutes* (*Bacilli*) | *Bacillus simplex* (AJ439078) | 99.8 |
| CB-281438 | JX840995 | *Firmicutes* (*Bacilli*) | *Bacillus simplex* (AJ439078) | 99.9 |
| CB-281428 | JX840987 | *Firmicutes* (*Bacilli*) | *Bacillus sporothermodurans* (U49078) | 99.3 |
| CB-281422 | JX840981 | *Firmicutes* (*Bacilli*) | *Paenibacillus alginolyticus* (AB073362) | 98.5 |
| CB-281421 | JX840980 | *Firmicutes* (*Bacilli*) | *Paenibacillus alginolyticus* (AB073362) | 98.9 |
| CB-281423 | JX840982 | *Firmicutes* (*Bacilli*) | *Paenibacillus lautus* (AB073188) | 98.9 |
| CB-281536 | JX841071 | *Firmicutes* (*Bacilli*) | *Paenibacillus lautus* (AB073188) | 99.6 |
| CB-281537 | JX841072 | *Firmicutes* (*Bacilli*) | *Paenibacillus pabuli* (AB045094) | 99.0 |
| CB-281424 | JX840983 | *Firmicutes* (*Bacilli*) | *Psychrobacillus psychrodurans* (AJ277984) | 99.6 |
| CB-281509 | JX841047 | *Proteobacteria* (*Alphaproteobacteria*) | *Ancylobacter polymorphus* (AY211516) | 97.3 |
| CB-281528 | JX841063 | *Proteobacteria* (*Alphaproteobacteria*) | *Devosia neptuniae* (AF469072) | 99.6 |
| CB-281530 | JX841065 | *Proteobacteria* (*Alphaproteobacteria*) | *Devosia neptuniae* (AF469072) | 99.7 |
| CB-281529 | JX841064 | *Proteobacteria* (*Alphaproteobacteria*) | *Devosia neptuniae* (AF469072) | 99.8 |
| CB-281495 | JX841033 | *Proteobacteria* (*Alphaproteobacteria*) | *Methylobacterium brachiatum* (AB175649) | 99.9 |
| CB-281531 | JX841066 | *Proteobacteria* (*Alphaproteobacteria*) | *Methylobacterium marchantiae* (FJ157976) | 95.5 |
| CB-281496 | JX841034 | *Proteobacteria* (*Alphaproteobacteria*) | *Methylobacterium marchantiae* (FJ157976) | 99.9 |
| CB-281497 | JX841035 | *Proteobacteria* (*Alphaproteobacteria*) | *Methylobacterium oxalidis* (AB607860) | 96.5 |
| CB-281498 | JX841036 | *Proteobacteria* (*Alphaproteobacteria*) | *Microvirga aerilata* (GQ421849) | 98.2 |
| CB-281510 | JX841048 | *Proteobacteria* (*Alphaproteobacteria*) | *Phyllobacterium ifriqiyense* (AY785325) | 98.5 |
| CB-281532 | JX841067 | *Proteobacteria* (*Alphaproteobacteria*) | *Phyllobacterium ifriqiyense* (AY785325) | 99.3 |
| CB-281499 | JX841037 | *Proteobacteria* (*Alphaproteobacteria*) | *Phyllobacterium ifriqiyense* (AY785325) | 99.5 |
| CB-281500 | JX841038 | *Proteobacteria* (*Alphaproteobacteria*) | *Phyllobacterium ifriqiyense* (AY785325) | 99.5 |
| CB-281533 | JX841068 | *Proteobacteria* (*Alphaproteobacteria*) | *Phyllobacterium ifriqiyense* (AY785325) | 99.8 |
| CB-281550 | JX841085 | *Proteobacteria* (*Alphaproteobacteria*) | *Phyllobacterium trifolii* (AY786080) | 99.4 |
| CB-281501 | JX841039 | *Proteobacteria* (*Alphaproteobacteria*) | *Phyllobacterium trifolii* (AY786080) | 99.5 |
| CB-281502 | JX841040 | *Proteobacteria* (*Alphaproteobacteria*) | *Phyllobacterium trifolii* (AY786080) | 99.6 |
| CB-281511 | JX841049 | *Proteobacteria* (*Alphaproteobacteria*) | *Phyllobacterium trifolii* (AY786080) | 99.7 |
| CB-281513 | JX841051 | *Proteobacteria* (*Alphaproteobacteria*) | *Phyllobacterium trifolii* (AY786080) | 99.7 |
| CB-281553 | JX841088 | *Proteobacteria* (*Alphaproteobacteria*) | *Phyllobacterium trifolii* (AY786080) | 99.8 |
| CB-281503 | JX841041 | *Proteobacteria* (*Alphaproteobacteria*) | *Phyllobacterium trifolii* (AY786080) | 99.9 |
| CB-281512 | JX841050 | *Proteobacteria* (*Alphaproteobacteria*) | *Phyllobacterium trifolii* (AY786080) | 99.9 |
| CB-281514 | JX841052 | *Proteobacteria* (*Alphaproteobacteria*) | *Phyllobacterium trifolii* (AY786080) | 99.9 |
| CB-281551 | JX841086 | *Proteobacteria* (*Alphaproteobacteria*) | *Phyllobacterium trifolii* (AY786080) | 99.9 |
| CB-281552 | JX841087 | *Proteobacteria* (*Alphaproteobacteria*) | *Phyllobacterium trifolii* (AY786080) | 99.9 |
| CB-281508 | JX841046 | *Proteobacteria* (*Alphaproteobacteria*) | *Rhizobium fabae* (DQ835306) | 99.0 |
| CB-281505 | JX841043 | *Proteobacteria* (*Alphaproteobacteria*) | *Rhizobium giardinii* (U86344) | 97.6 |
| CB-281506 | JX841044 | *Proteobacteria* (*Alphaproteobacteria*) | *Rhizobium giardinii* (U86344) | 98.6 |
| CB-281504 | JX841042 | *Proteobacteria* (*Alphaproteobacteria*) | *Rhizobium leguminosarum* (U29386) | 98.3 |
| CB-281507 | JX841045 | *Proteobacteria* (*Alphaproteobacteria*) | *Rhizobium leguminosarum* (U29386) | 99.1 |
| CB-282242 | JX841089 | *Proteobacteria* (*Alphaproteobacteria*) | *Skermanella aerolata* (DQ672568) | 98.9 |
| CB-281515 | JX841053 | *Proteobacteria* (*Alphaproteobacteria*) | *Sphingomonas faeni* (AJ429239) | 99.9 |
| CB-281516 | JX841054 | *Proteobacteria* (*Alphaproteobacteria*) | *Sphingomonas faeni* (AJ429239) | 99.9 |
| CB-281493 | JX841031 | *Proteobacteria* (*Betaproteobacteria*) | *Burkholderia graminis* (U96939) | 99.1 |
| CB-281494 | JX841032 | *Proteobacteria* (*Betaproteobacteria*) | *Burkholderia graminis* (U96939) | 99.7 |
| CB-281517 | JX841055 | *Proteobacteria* (*Gammaproteobacteria*) | *Acinetobacter lwoffii* (X81665) | 100 |
| CB-281538 | JX841073 | *Proteobacteria* (*Gammaproteobacteria*) | *Pseudomonas reinekei* (AM293565) | 98.5 |
| CB-281442 | JX840999 | *Proteobacteria* (*Gammaproteobacteria*) | *Stenotrophomonas rhizophila* (AJ293463) | 99.8 |
| CB-281441 | JX840998 | *Proteobacteria* (*Gammaproteobacteria*) | *Stenotrophomonas rhizophila* (AJ293463) | 99.9 |
| CB-281443 | JX841000 | *Proteobacteria* (*Gammaproteobacteria*) | *Stenotrophomonas rhizophila* (AJ293463) | 99.9 |
| CB-281444 | JX841001 | *Proteobacteria* (*Gammaproteobacteria*) | *Xanthomonas arboricola* (Y10757) | 99.3 |

^a^In parenthesis the class level is indicated except for representatives of *Actinobacteria* where the subclass level is shown.
