# Supplementary material for: Assessing Bacterial Diversity in the Rhizosphere of Thymus zygis Growing in the Sierra Nevada National Park (Spain) through Culture-Dependent and Independent Approaches
Source: PLoS One. 2016 Jan 7;11(1):e0146558. doi: 10.1371/journal.pone.0146558 (PMC4711807; doi:10.1371/journal.pone.0146558)
Supplement: S3 Table — (DOCX) [file pone.0146558.s006.docx]

**Table S3.** Taxonomic diversity of clone library based on their 16S rRNA gene sequences.

| **Clone** | **Accession number** | **Phylum (Class)^a^** | **Closet type strain^a^** | **Similarity (%)** |
| --- | --- | --- | --- | --- |
| SNNP_2012_13 | JX114346 | *Acidobacteria* (*Acidobacteria_Gp1*) | *Acidicapsa borealis* (FR774763) | 83.9 |
| SNNP_2012_47 | JX114380 | *Acidobacteria* (*Acidobacteria_Gp1*) | *Acidicapsa borealis* (FR774763) | 84.0 |
| SNNP_2012_147 | JX114480 | *Acidobacteria* (*Acidobacteria_Gp1*) | *Acidicapsa borealis* (FR774763) | 84.1 |
| SNNP_2012_121 | JX114454 | *Acidobacteria* (*Acidobacteria_Gp1*) | *Acidicapsa borealis* (FR774763) | 84.2 |
| SNNP_2012_160 | JX114493 | *Acidobacteria* (*Acidobacteria_Gp1*) | *Acidicapsa borealis* (FR774763) | 84.2 |
| SNNP_2012_177 | JX114509 | *Acidobacteria* (*Acidobacteria_Gp1*) | *Acidicapsa borealis* (FR774763) | 84.2 |
| SNNP_2012_22 | JX114355 | *Acidobacteria* (*Acidobacteria_Gp1*) | *Acidicapsa borealis* (FR774763) | 84.2 |
| SNNP_2012_75 | JX114408 | *Acidobacteria* (*Acidobacteria_Gp1*) | *Acidicapsa borealis* (FR774763) | 84.3 |
| SNNP_2012_132 | JX114465 | *Acidobacteria* (*Acidobacteria_Gp1*) | *Acidicapsa borealis* (FR774763) | 84.4 |
| SNNP_2012_170 | JX114503 | *Acidobacteria* (*Acidobacteria_Gp1*) | *Acidicapsa borealis* (FR774763) | 84.4 |
| SNNP_2012_2 | JX114335 | *Acidobacteria* (*Acidobacteria_Gp1*) | *Acidicapsa borealis* (FR774763) | 84.4 |
| SNNP_2012_144 | JX114477 | *Acidobacteria* (*Acidobacteria_Gp1*) | *Acidicapsa borealis* (FR774763) | 84.5 |
| SNNP_2012_156 | JX114489 | *Acidobacteria* (*Acidobacteria_Gp1*) | *Acidicapsa borealis* (FR774763) | 84.5 |
| SNNP_2012_19 | JX114352 | *Acidobacteria* (*Acidobacteria_Gp1*) | *Acidicapsa borealis* (FR774763) | 84.5 |
| SNNP_2012_81 | JX114414 | *Acidobacteria* (*Acidobacteria_Gp1*) | *Acidicapsa borealis* (FR774763) | 84.5 |
| SNNP_2012_130 | JX114463 | *Acidobacteria* (*Acidobacteria_Gp1*) | *Acidicapsa borealis* (FR774763) | 84.6 |
| SNNP_2012_119 | JX114452 | *Acidobacteria* (*Acidobacteria_Gp1*) | *Acidicapsa borealis* (FR774763) | 84.7 |
| SNNP_2012_120 | JX114453 | *Acidobacteria* (*Acidobacteria_Gp1*) | *Acidicapsa borealis* (FR774763) | 84.7 |
| SNNP_2012_70 | JX114403 | *Acidobacteria* (*Acidobacteria_Gp1*) | *Acidicapsa borealis* (FR774763) | 84.7 |
| SNNP_2012_82 | JX114415 | *Acidobacteria* (*Acidobacteria_Gp1*) | *Acidicapsa borealis* (FR774763) | 84.7 |
| SNNP_2012_159 | JX114492 | *Acidobacteria* (*Acidobacteria_Gp1*) | *Acidicapsa borealis* (FR774763) | 84.8 |
| SNNP_2012_174 | JX114506 | *Acidobacteria* (*Acidobacteria_Gp1*) | *Acidicapsa borealis* (FR774763) | 84.8 |
| SNNP_2012_5 | JX114338 | *Acidobacteria* (*Acidobacteria_Gp1*) | *Acidicapsa borealis* (FR774763) | 84.8 |
| SNNP_2012_139 | JX114472 | *Acidobacteria* (*Acidobacteria_Gp1*) | *Acidicapsa borealis* (FR774763) | 84.9 |
| SNNP_2012_178 | JX114510 | *Acidobacteria* (*Acidobacteria_Gp1*) | *Acidicapsa borealis* (FR774763) | 85.1 |
| SNNP_2012_28 | JX114361 | *Acidobacteria* (*Acidobacteria_Gp1*) | *Acidicapsa ligni* (DQ528761) | 84.5 |
| SNNP_2012_155 | JX114488 | *Acidobacteria* (*Acidobacteria_Gp1*) | *Acidicapsa ligni* (DQ528761) | 84.7 |
| SNNP_2012_95 | JX114428 | *Acidobacteria* (*Acidobacteria_Gp1*) | *Acidicapsa ligni* (DQ528761) | 84.9 |
| SNNP_2012_52 | JX114385 | *Acidobacteria* (*Acidobacteria_Gp1*) | *Acidicapsa ligni* (DQ528761) | 85.0 |
| SNNP_2012_1 | JX114334 | *Acidobacteria* (*Acidobacteria_Gp1*) | *Acidicapsa ligni* (DQ528761) | 85.3 |
| SNNP_2012_32 | JX114365 | *Acidobacteria* (*Acidobacteria_Gp1*) | *Acidicapsa ligni* (DQ528761) | 85.3 |
| SNNP_2012_42 | JX114375 | *Acidobacteria* (*Acidobacteria_Gp1*) | *Acidicapsa ligni* (DQ528761) | 85.3 |
| SNNP_2012_24 | JX114357 | *Acidobacteria* (*Acidobacteria_Gp1*) | *Acidobacterium capsulatum* (CP001472) | 85.0 |
| SNNP_2012_56 | JX114389 | *Acidobacteria* (*Acidobacteria_Gp1*) | *Acidobacterium capsulatum* (CP001472) | 85.0 |
| SNNP_2012_138 | JX114471 | *Acidobacteria* (*Acidobacteria_Gp1*) | *Acidobacterium capsulatum* (CP001472) | 92.6 |
| SNNP_2012_145 | JX114478 | *Acidobacteria* (*Acidobacteria_Gp1*) | *Acidobacterium capsulatum* (CP001472) | 92.6 |
| SNNP_2012_154 | JX114487 | *Acidobacteria* (*Acidobacteria_Gp1*) | *Edaphobacter aggregans* (DQ528761) | 85.9 |
| SNNP_2012_35 | JX114368 | *Acidobacteria* (*Acidobacteria_Gp1*) | *Edaphobacter modestus* (DQ528760) | 92.7 |
| SNNP_2012_158 | JX114491 | *Acidobacteria* (*Acidobacteria_Gp1*) | *Granulicella pectinivorans* (AM887757) | 85.4 |
| SNNP_2012_167 | JX114500 | *Acidobacteria* (*Acidobacteria_Gp1*) | *Granulicella pectinivorans* (AM887757) | 85.4 |
| SNNP_2012_91 | JX114424 | *Acidobacteria* (*Acidobacteria_Gp1*) | *Granulicella tundricola* (HQ687088) | 84.8 |
| SNNP_2012_15 | JX114348 | *Acidobacteria* (*Acidobacteria_Gp1*) | *Telmatobacter bradus* (AM887760) | 84.4 |
| SNNP_2012_64 | JX114397 | *Acidobacteria* (*Acidobacteria_Gp1*) | *Terriglobus tenax* (JN543507) | 91.9 |
| SNNP_2012_134 | JX114467 | *Acidobacteria* (*Acidobacteria_Gp4*) | *Blastocatella fastidiosa* (JQ309130) | 88.7 |
| SNNP_2012_100 | JX114433 | *Acidobacteria* (*Acidobacteria_Gp4*) | *Blastocatella fastidiosa* (JQ309130) | 89.0 |
| SNNP_2012_14 | JX114347 | *Acidobacteria* (*Acidobacteria_Gp4*) | *Blastocatella fastidiosa* (JQ309130) | 90.1 |
| SNNP_2012_62 | JX114395 | *Acidobacteria* (*Acidobacteria_Gp4*) | *Blastocatella fastidiosa* (JQ309130) | 90.4 |
| SNNP_2012_63 | JX114396 | *Acidobacteria* (*Acidobacteria_Gp4*) | *Blastocatella fastidiosa* (JQ309130) | 90.4 |
| SNNP_2012_187 | JX114518 | *Acidobacteria* (*Acidobacteria_Gp4*) | *Blastocatella fastidiosa* (JQ309130) | 90.5 |
| SNNP_2012_67 | JX114400 | *Acidobacteria* (*Acidobacteria_Gp4*) | *Blastocatella fastidiosa* (JQ309130) | 92.5 |
| SNNP_2012_74 | JX114407 | *Acidobacteria* (*Acidobacteria_Gp4*) | *Blastocatella fastidiosa* (JQ309130) | 92.7 |
| SNNP_2012_90 | JX114423 | *Acidobacteria* (*Acidobacteria_Gp4*) | *Blastocatella fastidiosa* (JQ309130) | 92.7 |
| SNNP_2012_49 | JX114382 | *Acidobacteria* (*Acidobacteria_Gp4*) | *Blastocatella fastidiosa* (JQ309130) | 92.9 |
| SNNP_2012_50 | JX114383 | *Acidobacteria* (*Acidobacteria_Gp4*) | *Blastocatella fastidiosa* (JQ309130) | 93.0 |
| SNNP_2012_36 | JX114369 | *Acidobacteria* (*Acidobacteria_Gp4*) | *Blastocatella fastidiosa* (JQ309130) | 93.5 |
| SNNP_2012_55 | JX114388 | *Acidobacteria* (*Acidobacteria_Gp4*) | *Blastocatella fastidiosa* (JQ309130) | 93.5 |
| SNNP_2012_58 | JX114391 | *Acidobacteria* (*Acidobacteria_Gp4*) | *Blastocatella fastidiosa* (JQ309130) | 93.5 |
| SNNP_2012_72 | JX114405 | *Acidobacteria* (*Acidobacteria_Gp4*) | *Blastocatella fastidiosa* (JQ309130) | 93.5 |
| SNNP_2012_98 | JX114431 | *Acidobacteria* (*Acidobacteria_Gp4*) | *Blastocatella fastidiosa* (JQ309130) | 93.5 |
| SNNP_2012_27 | JX114360 | *Acidobacteria* (*Acidobacteria_Gp4*) | *Blastocatella fastidiosa* (JQ309130) | 93.6 |
| SNNP_2012_4 | JX114337 | *Acidobacteria* (*Acidobacteria_Gp4*) | *Blastocatella fastidiosa* (JQ309130) | 93.6 |
| SNNP_2012_141 | JX114474 | *Acidobacteria* (*Acidobacteria_Gp4*) | *Blastocatella fastidiosa* (JQ309130) | 93.7 |
| SNNP_2012_149 | JX114482 | *Acidobacteria* (*Acidobacteria_Gp4*) | *Blastocatella fastidiosa* (JQ309130) | 93.7 |
| SNNP_2012_118 | JX114451 | *Acidobacteria* (*Acidobacteria_Gp4*) | *Blastocatella fastidiosa* (JQ309130) | 93.8 |
| SNNP_2012_25 | JX114358 | *Acidobacteria* (*Acidobacteria_Gp4*) | *Blastocatella fastidiosa* (JQ309130) | 93.8 |
| SNNP_2012_83 | JX114416 | *Acidobacteria* (*Acidobacteria_Gp4*) | *Blastocatella fastidiosa* (JQ309130) | 93.9 |
| SNNP_2012_175 | JX114507 | *Acidobacteria* (*Acidobacteria_Gp4*) | *Blastocatella fastidiosa* (JQ309130) | 96.7 |
| SNNP_2012_106 | JX114439 | *Acidobacteria* (*Holophagae*) | *Geothrix fermentans* (U41563) | 83.4 |
| SNNP_2012_143 | JX114476 | *Acidobacteria* (*Holophagae*) | *Geothrix fermentans* (U41563) | 84.1 |
| SNNP_2012_150 | JX114483 | *Acidobacteria* (*Holophagae*) | *Holophaga foetida* (X77215) | 84.0 |
| SNNP_2012_104 | JX114437 | *Acidobacteria* (*Holophagae*) | *Holophaga foetida* (X77215) | 84.2 |
| SNNP_2012_88 | JX114421 | *Acidobacteria* (*Holophagae*) | *Holophaga foetida* (X77215) | 84.2 |
| SNNP_2012_99 | JX114432 | *Acidobacteria* (*Holophagae*) | *Holophaga foetida* (X77215) | 84.2 |
| SNNP_2012_46 | JX114379 | *Acidobacteria* (*Holophagae*) | *Holophaga foetida* (X77215) | 84.4 |
| SNNP_2012_109 | JX114442 | *Acidobacteria* (*Holophagae*) | *Holophaga foetida* (X77215) | 84.5 |
| SNNP_2012_165 | JX114498 | *Acidobacteria* (*Holophagae*) | *Holophaga foetida* (X77215) | 84.5 |
| SNNP_2012_23 | JX114356 | *Acidobacteria* (*Holophagae*) | *Holophaga foetida* (X77215) | 84.6 |
| SNNP_2012_3 | JX114336 | *Acidobacteria* (*Holophagae*) | *Holophaga foetida* (X77215) | 84.6 |
| SNNP_2012_166 | JX114499 | *Acidobacteria* (*Holophagae*) | *Holophaga foetida* (X77215) | 84.9 |
| SNNP_2012_108 | JX114441 | *Acidobacteria* (*Holophagae*) | *Holophaga foetida* (X77215) | 85.0 |
| SNNP_2012_48 | JX114381 | *Acidobacteria* (*Holophagae*) | *Holophaga foetida* (X77215) | 85.1 |
| SNNP_2012_80 | JX114413 | *Acidobacteria* (*Holophagae*) | *Holophaga foetida* (X77215) | 85.1 |
| SNNP_2012_87 | JX114420 | *Acidobacteria* (*Holophagae*) | *Holophaga foetida* (X77215) | 85.1 |
| SNNP_2012_61 | JX114394 | *Acidobacteria* (*Holophagae*) | *Holophaga foetida* (X77215) | 85.3 |
| SNNP_2012_168 | JX114501 | *Acidobacteria* (*Holophagae*) | *Holophaga foetida* (X77215) | 85.4 |
| SNNP_2012_31 | JX114364 | *Acidobacteria* (*Holophagae*) | *Holophaga foetida* (X77215) | 85.6 |
| SNNP_2012_135 | JX114468 | *Acidobacteria* (*Holophagae*) | *Holophaga foetida* (X77215) | 85.9 |
| SNNP_2012_146 | JX114479 | *Acidobacteria* (*Holophagae*) | *Holophaga foetida* (X77215) | 85.9 |
| SNNP_2012_76 | JX114409 | *Actinobacteria* (*Acidimicrobidae*) | *Aciditerrimonas ferrireducens* (AB517669) | 91.7 |
| SNNP_2012_12 | JX114345 | *Actinobacteria* (*Acidimicrobidae*) | *Ilumatobacter fluminis* (AB360343) | 92.7 |
| SNNP_2012_40 | JX114373 | *Actinobacteria* (*Acidimicrobidae*) | *Ilumatobacter fluminis* (AB360343) | 93.3 |
| SNNP_2012_41 | JX114374 | *Actinobacteria* (*Acidimicrobidae*) | *Ilumatobacter fluminis* (AB360343) | 93.3 |
| SNNP_2012_128 | JX114461 | *Actinobacteria* (*Rubrobacteridae*) | *Gaiella occulta* (JF423906) | 93.8 |
| SNNP_2012_152 | JX114485 | *Actinobacteria* (*Rubrobacteridae*) | *Gaiella occulta* (JF423906) | 93.8 |
| SNNP_2012_43 | JX114376 | *Actinobacteria* (*Rubrobacteridae*) | *Gaiella occulta* (JF423906) | 94.0 |
| SNNP_2012_34 | JX114367 | *Armatimonadetes* (*Fimbriimonadia*) | *Fimbriimonas ginsengisoli* (GQ339893) | 83.6 |
| SNNP_2012_136 | JX114469 | *Bacteroidetes* (*Bacteroidetes_incertae_sedis*) | *Ohtaekwangia koreensis* (GU117702) | 90.7 |
| SNNP_2012_122 | JX114455 | *Bacteroidetes* (*Bacteroidetes_incertae_sedis*) | *Ohtaekwangia koreensis* (GU117702) | 92.4 |
| SNNP_2012_116 | JX114449 | *Bacteroidetes* (*Bacteroidetes_incertae_sedis*) | *Ohtaekwangia koreensis* (GU117702) | 92.5 |
| SNNP_2012_65 | JX114398 | *Bacteroidetes* (*Flavobacteriia*) | *Flavobacterium frigidimaris* (AB183888) | 98.6 |
| SNNP_2012_37 | JX114370 | *Bacteroidetes* (*Flavobacteriia*) | *Flavobacterium granuli* (AB180738) | 95.6 |
| SNNP_2012_69 | JX114402 | *Bacteroidetes* (*Flavobacteriia*) | *Flavobacterium granuli* (AB180738) | 95.6 |
| SNNP_2012_29 | JX114362 | *Bacteroidetes* (*Flavobacteriia*) | *Flavobacterium koreense* (GU295967) | 97.5 |
| SNNP_2012_30 | JX114363 | *Bacteroidetes* (*Flavobacteriia*) | *Flavobacterium koreense* (GU295967) | 97.5 |
| SNNP_2012_45 | JX114378 | *Bacteroidetes* (*Flavobacteriia*) | *Flavobacterium subsaxonicum* (AM934666) | 96.7 |
| SNNP_2012_93 | JX114426 | *Bacteroidetes* (*Sphingobacteriia*) | *Chitinophaga niabensis* (EU714259) | 92.0 |
| SNNP_2012_105 | JX114438 | *Bacteroidetes* (*Sphingobacteriia*) | *Chitinophaga sancti* (AB078066) | 90.1 |
| SNNP_2012_182 | JX114513 | *Bacteroidetes* (*Sphingobacteriia*) | *Ferruginibacter lapsinanis* (FJ177532) | 93.2 |
| SNNP_2012_112 | JX114445 | *Bacteroidetes* (*Sphingobacteriia*) | *Ferruginibacter lapsinanis* (FJ177532) | 93.5 |
| SNNP_2012_113 | JX114446 | *Bacteroidetes* (*Sphingobacteriia*) | *Ferruginibacter lapsinanis* (FJ177532) | 93.5 |
| SNNP_2012_53 | JX114386 | *Bacteroidetes* (*Sphingobacteriia*) | *Ferruginibacter lapsinanis* (FJ177532) | 94.8 |
| SNNP_2012_89 | JX114422 | *Bacteroidetes* (*Sphingobacteriia*) | *Ferruginibacter lapsinani*s (FJ177532) | 94.9 |
| SNNP_2012_171 | JX114504 | *Bacteroidetes* (*Sphingobacteriia*) | *Ferruginibacter lapsinanis* (FJ177532) | 96.1 |
| SNNP_2012_38 | JX114371 | *Bacteroidetes* (*Sphingobacteriia*) | *Ferruginibacter lapsinanis* (FJ177532) | 96.1 |
| SNNP_2012_97 | JX114430 | *Bacteroidetes* (*Sphingobacteriia*) | *Flavihumibacter petaseus* (EU854577) | 94.3 |
| SNNP_2012_92 | JX114425 | *Bacteroidetes* (*Sphingobacteriia*) | *Flavisolibacter ginsengisoli* (AB267477) | 96.8 |
| SNNP_2012_79 | JX114412 | *Bacteroidetes* (*Sphingobacteriia*) | *Flavisolibacter ginsengiterrae* (AB267476) | 97.1 |
| SNNP_2012_20 | JX114353 | *Bacteroidetes* (*Sphingobacteriia*) | *Flavisolibacter ginsengiterrae* (AB267476) | 97.4 |
| SNNP_2012_54 | JX114387 | *Bacteroidetes* (*Sphingobacteriia*) | *Flavitalea populi* (HM130561) | 98.1 |
| SNNP_2012_107 | JX114440 | *Bacteroidetes* (*Sphingobacteriia*) | *Haliscomenobacter hydrossis* (AJ784892) | 88.7 |
| SNNP_2012_125 | JX114458 | *Bacteroidetes* (*Sphingobacteriia*) | *Lewinella marina* (AB301495) | 86.5 |
| SNNP_2012_117 | JX114450 | *Bacteroidetes* (*Sphingobacteriia*) | *Mucilaginibacter dorajii* (GU139697) | 98.0 |
| SNNP_2012_115 | JX114448 | *Bacteroidetes* (*Sphingobacteriia*) | *Mucilaginibacter gracilis* (AM490403) | 98.5 |
| SNNP_2012_148 | JX114481 | *Bacteroidetes* (*Sphingobacteriia*) | *Pedobacter composti* (AB267720) | 95.6 |
| SNNP_2012_8 | JX114341 | *Bacteroidetes* (*Sphingobacteriia*) | *Pedobacter ginsengisoli* (AB245371) | 99.1 |
| SNNP_2012_103 | JX114436 | *Bacteroidetes* (*Sphingobacteriia*) | *Pedobacter ginsengisoli* (AB245371) | 99.2 |
| SNNP_2012_44 | JX114377 | *Bacteroidetes* (*Sphingobacteriia*) | *Pedobacter oryzae* (EU109726) | 98.1 |
| SNNP_2012_114 | JX114447 | *Bacteroidetes* (*Sphingobacteriia*) | *Pedobacter ruber* (HQ882803) | 95.6 |
| SNNP_2012_186 | JX114517 | *Bacteroidetes* (*Sphingobacteriia*) | *Pedobacter sandarakinus* (DQ235228) | 84.2 |
| SNNP_2012_140 | JX114473 | *Bacteroidetes* (*Sphingobacteriia*) | *Pedobacter sandarakinus* (DQ235228) | 84.8 |
| SNNP_2012_126 | JX114459 | *Bacteroidetes* (*Sphingobacteriia*) | *Pedobacter soli* (AM279215) | 85.6 |
| SNNP_2012_66 | JX114399 | *Bacteroidetes* (*Sphingobacteriia*) | *Segetibacter aerophilus* (GQ421847) | 85.9 |
| SNNP_2012_124 | JX114457 | *Bacteroidetes* (*Sphingobacteriia*) | *Segetibacter aerophilus* (GQ421847) | 94.8 |
| SNNP_2012_77 | JX114410 | *Bacteroidetes* (*Sphingobacteriia*) | *Segetibacter aerophilus* (GQ421847) | 97.5 |
| SNNP_2012_161 | JX114494 | *Bacteroidetes* (*Sphingobacteriia*) | *Solitalea koreensis* (EU787448) | 89.9 |
| SNNP_2012_173 | JX114505 | *Bacteroidetes* (*Sphingobacteriia*) | *Solitalea koreensis* (EU787448) | 89.9 |
| SNNP_2012_33 | JX114366 | *Firmicutes* (*Bacilli*) | *Cohnella fontinalis* (AB362828) | 77.3 |
| SNNP_2012_169 | JX114502 | *Firmicutes* (*Bacilli*) | *Exiguobacterium aurantiacum* (DQ019166) | 78.2 |
| SNNP_2012_111 | JX114444 | *Firmicutes* (*Bacilli*) | *Exiguobacterium undae* (DQ019165) | 80.9 |
| SNNP_2012_7 | JX114340 | *Firmicutes* (*Bacilli*) | *Pirellula staleyi* (AJ231183) | 92.0 |
| SNNP_2012_86 | JX114419 | *Firmicutes* (*Bacilli*) | *Pirellula staleyi* (AJ231183) | 92.3 |
| SNNP_2012_176 | JX114508 | *Firmicutes* (*Bacilli*) | *Staphylococcus rostri* (FM242137) | 78.4 |
| SNNP_2012_131 | JX114464 | *Firmicutes* (*Clostridia*) | *Heliobacterium undosum* (AF249679) | 78.8 |
| SNNP_2012_85 | JX114418 | *Firmicutes* (*Clostridia*) | *Moorella humiferrea* (GQ872425) | 81.0 |
| SNNP_2012_26 | JX114359 | *Firmicutes* (*Clostridia*) | *Thermacetogenium phaeum* (AB020336) | 83.1 |
| SNNP_2012_96 | JX114429 | *Firmicutes* (*Thermolithobacteria*) | *Thermolithobacter carboxydivorans* (DQ095862) | 84.0 |
| SNNP_2012_51 | JX114384 | *Firmicutes* (*Thermolithobacteria*) | *Thermolithobacter carboxydivorans* (DQ095862) | 84.1 |
| SNNP_2012_60 | JX114393 | *Ignavibacteriae* (*Ignavibacteria*) | *Ignavibacterium album* (AB478415) | 85.3 |
| SNNP_2012_11 | JX114344 | *Nitrospira* (*Nitrospira*) | *Nitrospira moscoviensis* (X82558) | 88.9 |
| SNNP_2012_157 | JX114490 | *Nitrospira* (*Nitrospira*) | *Nitrospira moscoviensis* (X82558) | 94.7 |
| SNNP_2012_129 | JX114462 | *Planctomycetes* (*Phycisphaerae*) | *Phycisphaera mikurensis* (AB447464) | 81.6 |
| SNNP_2012_123 | JX114456 | *Planctomycetes* (*Planctomycetia*) | *Gemmata obscuriglobus* (X56305) | 91.1 |
| SNNP_2012_163 | JX114496 | *Planctomycetes* (*Planctomycetia*) | *Schlesneria paludicola* (AM162407) | 83.4 |
| SNNP_2012_162 | JX114495 | *Proteobacteria* (*Alphaproteobacteria*) | *Beijerinckia indica* (AJ563930) | 96.9 |
| SNNP_2012_153 | JX114486 | *Proteobacteria* (*Alphaproteobacteria*) | *Caulobacter segnis* (AB023427) | 96.7 |
| SNNP_2012_17 | JX114350 | *Proteobacteria* (*Alphaproteobacteria*) | *Inquilinus ginsengisoli* (AB245352) | 86.3 |
| SNNP_2012_6 | JX114339 | *Proteobacteria* (*Alphaproteobacteria*) | *Inquilinus ginsengisoli* (AB245352) | 86.4 |
| SNNP_2012_142 | JX114475 | *Proteobacteria* (*Alphaproteobacteria*) | *Mesorhizobium metallidurans* (AM930381) | 99.9 |
| SNNP_2012_94 | JX114427 | *Proteobacteria* (*Alphaproteobacteria*) | *Pseudolabrys taiwanensis* (DQ062742) | 94.6 |
| SNNP_2012_137 | JX114470 | *Proteobacteria* (*Alphaproteobacteria*) | *Rhizobium giardinii* (U86344) | 99.2 |
| SNNP_2012_110 | JX114443 | *Proteobacteria* (*Alphaproteobacteria*) | *Sphingobium lactosutens* (EU675846) | 96.9 |
| SNNP_2012_180 | JX114512 | *Proteobacteria* (*Alphaproteobacteria*) | *Sphingomonas asaccharolytica* (Y09639) | 98.4 |
| SNNP_2012_164 | JX114497 | *Proteobacteria* (*Alphaproteobacteria*) | *Tardiphaga robiniae* (FR753034) | 99.6 |
| SNNP_2012_188 | JX114519 | *Proteobacteria* (*Betaproteobacteria*) | *Azonexus fungiphilus* (AF011350) | 92.7 |
| SNNP_2012_71 | JX114404 | *Proteobacteria* (*Betaproteobacteria*) | *Burkholderia graminis* (U96939) | 99.8 |
| SNNP_2012_21 | JX114354 | *Proteobacteria* (*Betaproteobacteria*) | *Denitratisoma oestradiolicum* (AY879297) | 90.8 |
| SNNP_2012_102 | JX114435 | *Proteobacteria* (*Betaproteobacteria*) | *Denitratisoma oestradiolicum* (AY879297) | 92.1 |
| SNNP_2012_151 | JX114484 | *Proteobacteria* (*Betaproteobacteria*) | *Duganella phyllosphaerae* (FR852575) | 98.6 |
| SNNP_2012_184 | JX114515 | *Proteobacteria* (*Betaproteobacteria*) | *Massilia jejuensis* (FJ969486) | 98.0 |
| SNNP_2012_10 | JX114343 | *Proteobacteria* (*Betaproteobacteria*) | *Methylotenera mobilis* (DQ287786) | 98.8 |
| SNNP_2012_16 | JX114349 | *Proteobacteria* (*Betaproteobacteria*) | *Nitrosospira multiformis* (CP000103) | 91.3 |
| SNNP_2012_179 | JX114511 | *Proteobacteria* (*Betaproteobacteria*) | *Nitrosospira multiformis* (CP000103) | 91.5 |
| SNNP_2012_185 | JX114516 | *Proteobacteria* (*Betaproteobacteria*) | *Noviherbaspirillum soli* (HQ830498) | 97.4 |
| SNNP_2012_78 | JX114411 | *Proteobacteria* (*Betaproteobacteria*) | *Noviherbaspirillum soli* (HQ830498) | 97.4 |
| SNNP_2012_18 | JX114351 | *Proteobacteria* (*Betaproteobacteria*) | *Variovorax boronicumulans* (AB300597) | 99.6 |
| SNNP_2012_57 | JX114390 | *Proteobacteria* (*Deltaproteobacteria*) | *Cystobacter miniatus* (DQ768111) | 93.9 |
| SNNP_2012_73 | JX114406 | *Proteobacteria* (*Deltaproteobacteria*) | *Cystobacter miniatus* (DQ768111) | 93.9 |
| SNNP_2012_84 | JX114417 | *Proteobacteria* (*Gammaproteobacteria*) | *Arenimonas oryziterrae* (EU376961) | 97.9 |
| SNNP_2012_133 | JX114466 | *Proteobacteria* (*Gammaproteobacteria*) | *Ectothiorhodosinus mongolicus* (AY298904) | 90.7 |
| SNNP_2012_183 | JX114514 | *Proteobacteria* (*Gammaproteobacteria*) | *Luteimonas cucumeris* (HQ874629) | 98.9 |
| SNNP_2012_68 | JX114401 | *Proteobacteria* (*Gammaproteobacteria*) | *Rhizobacter dauci* (AB297965) | 96.7 |
| SNNP_2012_9 | JX114342 | *Proteobacteria* (*Gammaproteobacteria*) | *Rhizobacter dauci* (AB297965) | 98.3 |
| SNNP_2012_39 | JX114372 | *Proteobacteria* (*Gammaproteobacteria*) | *Steroidobacter denitrificans* (EF605262) | 91.2 |
| SNNP_2012_127 | JX114460 | *Proteobacteria* (*Gammaproteobacteria*) | *Steroidobacter denitrificans* (EF605262) | 97.2 |

^a^In parenthesis the class level is indicated except for representatives of *Actinobacteria* where the subclass level is shown.
