# Supplementary material for: Assessing Bacterial Diversity in the Rhizosphere of Thymus zygis Growing in the Sierra Nevada National Park (Spain) through Culture-Dependent and Independent Approaches
Source: PLoS One. 2016 Jan 7;11(1):e0146558. doi: 10.1371/journal.pone.0146558 (PMC4711807; doi:10.1371/journal.pone.0146558)
Supplement: S4 Table — 1, Cultured bacteria; 2, Clone library; 3, Pyrosequencing 2010; 4, Pyrosequencing 2010; 5, Core microbiome. (DOCX) [file pone.0146558.s007.docx]

| **KO categories** | **1** | **2** | **3** | **4** | **5** |
| --- | --- | --- | --- | --- | --- |
| **Metabolism** | **54.6** | **51.0** | **51.6** | **52.5** | **52.6** |
| **Carbohydrate metabolism** | **13.3** | **13.9** | **12.4** | **12.7** | **12.6** |
| ko00030 Pentose phosphate pathway | 0.9 | 0.9 | 0.9 | 0.9 | 0.9 |
| ko00051 Fructose and mannose metabolism | 1.3 | 1.4 | 1.2 | 1.3 | 1.2 |
| ko00500 Starch and sucrose metabolism | 2.3 | 2.6 | 1.8 | 1.9 | 1.9 |
| ko00520 Amino sugar and nucleotide sugar metabolism | 1.7 | 2.2 | 1.5 | 1.6 | 1.6 |
| ko00620 Pyruvate metabolism | 1.3 | 1.1 | 1.3 | 1.3 | 1.3 |
| ko00630 Glyoxylate and dicarboxylate metabolism | 1.3 | 1.2 | 1.4 | 1.4 | 1.4 |
| ko00650 Butanoate metabolism | 1.0 | 0.8 | 1.0 | 1.0 | 1.0 |
| ko00010 Glycolysis/Gluconeogenesis | 0.9 | 0.8 | 0.8 | 0.8 | 0.8 |
| ko00640 Propanoate metabolism | 0.7 | 0.5 | 0.7 | 0.7 | 0.7 |
| ko00020 Citrate cycle (TCA cycle) | 0.7 | 0.7 | 0.7 | 0.7 | 0.7 |
| ko00040 Pentose and glucuronate interconversions | 0.6 | 0.8 | 0.6 | 0.6 | 0.6 |
| ko00052 Galactose metabolism | 0.6 | 0.9 | 0.5 | 0.5 | 0.5 |
| **Lipid metabolism** | **2.7** | **2.0** | **2.3** | **2.4** | **2.4** |
| ko00061 Fatty acid biosynthesis | 1.0 | 0.7 | 0.9 | 0.9 | 0.9 |
| ko00561 Glycerolipid metabolism | 0.8 | 0.4 | 0.6 | 0.6 | 0.6 |
| ko00564 Glycerophospholipid metabolism | 0.9 | 0.9 | 0.8 | 0.9 | 0.9 |
| **Energy metabolism** | **6.3** | **6.8** | **6.6** | **6.7** | **6.7** |
| ko00190 Oxidative phosphorylation | 1.7 | 2.1 | 1.8 | 1.9 | 1.9 |
| ko00680 Methane metabolism | 1.4 | 1.1 | 1.3 | 1.3 | 1.3 |
| ko00720 Carbon fixation pathways in prokaryotes | 0.9 | 1.0 | 1.0 | 1.0 | 1.0 |
| ko00910 Nitrogen metabolism | 1.8 | 2.1 | 2.0 | 2.0 | 2.0 |
| ko00920 Sulfur metabolism | 0.5 | 0.5 | 0.5 | 0.5 | 0.5 |
| **Nucleotide metabolism** | **5.1** | **5.1** | **4.9** | **5.1** | **5.1** |
| ko00230 Purine metabolism | 3.1 | 3.1 | 3.0 | 3.1 | 3.1 |
| ko00240 Pyrimidine metabolism | 2.0 | 2.0 | 1.9 | 2.0 | 2.0 |
| **Amino acid metabolism** | **11.9** | **11.0** | **12.0** | **12.1** | **12.1** |
| ko00250 Alanine aspartate and glutamate metabolism | 1.1 | 1.1 | 1.0 | 1.0 | 1.0 |
| ko00260 Glycine serine and threonine metabolism | 1.6 | 1.5 | 1.6 | 1.6 | 1.6 |
| ko00270 Cysteine and methionine metabolism | 1.0 | 1.1 | 1.1 | 1.1 | 1.1 |
| ko00280 Valine leucine and isoleucine degradation | 1.0 | 0.8 | 1.0 | 1.0 | 1.0 |
| ko00300 Lysine biosynthesis | 1.0 | 0.8 | 0.9 | 0.9 | 0.9 |
| ko00330 Arginine and proline metabolism | 1.8 | 1.7 | 2.0 | 1.9 | 1.9 |
| ko00400 Phenylalanine tyrosine and tryptophan biosynthesis | 1.0 | 1.0 | 1.0 | 1.0 | 1.0 |
| ko00340 Histidine metabolism | 0.8 | 0.8 | 0.8 | 0.9 | 0.9 |
| ko00360 Phenylalanine metabolism | 0.8 | 0.5 | 0.8 | 0.8 | 0.8 |
| ko00350 Tyrosine metabolism | 0.7 | 0.5 | 0.7 | 0.7 | 0.7 |
| ko00310 Lysine degradation | 0.6 | 0.7 | 0.6 | 0.7 | 0.7 |
| ko00290 Valine, leucine and isoleucine biosynthesis | 0.5 | 0.5 | 0.5 | 0.5 | 0.5 |
| **Metabolism of other amino acids** | **1.4** | **1.3** | **1.4** | **1.3** | **1.4** |
| ko00480 Glutathione metabolism | 0.9 | 0.8 | 0.9 | 0.8 | 0.9 |
| ko00450 Selenocompound metabolism | 0.5 | 0.5 | 0.5 | 0.5 | 0.5 |
| **Glycan biosynthesis and metabolism** | **1.3** | **1.3** | **1.3** | **1.3** | **1.3** |
| ko00550 Peptidoglycan biosynthesis | 1.3 | 1.4 | 1.3 | 1.3 | 1.3 |
| **Metabolism of cofactors and vitamins** | **5.8** | **5.5** | **5.6** | **5.9** | **5.9** |
| ko00860 Porphyrin and chlorophyll metabolism | 1.8 | 1.6 | 1.8 | 1.8 | 1.8 |
| ko00130 Ubiquinone and other terpenoid-quinone biosynthesis | 0.8 | 0.7 | 0.8 | 0.8 | 0.8 |
| ko00760 Nicotinate and nicotinamide metabolism | 0.8 | 0.8 | 0.7 | 0.8 | 0.8 |
| ko00770 Pantothenate and CoA biosynthesis | 0.7 | 0.6 | 0.7 | 0.7 | 0.7 |
| ko00790 Folate biosynthesis | 0.6 | 0.5 | 0.5 | 0.6 | 0.6 |
| ko00780 Biotin metabolism | 0.6 | 0.7 | 0.6 | 0.7 | 0.7 |
| ko00730 Thiamine metabolism | 0.5 | 0.6 | 0.5 | 0.5 | 0.5 |
| **Metabolism of terpenoids and polyketides** | **4.6** | **2.7** | **3.2** | **3.2** | **3.3** |
| [ko01054 Nonribosomal peptide structures](http://www.genome.jp/kegg-bin/show_pathway?map=map01054&show_description=show) | 1.0 | 0.9 | 0.5 | 0.5 | 0.6 |
| ko00900 Terpenoid backbone biosynthesis | 0.9 | 0.8 | 0.8 | 0.8 | 0.8 |
| ko01053 Biosynthesis of siderophore group nonribosomal peptides | 0.8 | 0.3 | 0.5 | 0.5 | 0.5 |
| ko00281 Geraniol degradation | 0.7 | 0.3 | 0.6 | 0.6 | 0.6 |
| ko01051 Biosynthesis of ansamycins | 0.7 | 0.2 | 0.4 | 0.4 | 0.4 |
| ko00903 Limonene and pinene degradation | 0.5 | 0.2 | 0.4 | 0.4 | 0.4 |
| **Xenobiotics biodegradation and metabolism** | **2.1** | **1.3** | **1.9** | **1.8** | **1.8** |
| ko00362 Benzoate degradation | 0.9 | 0.5 | 0.8 | 0.7 | 0.7 |
| ko00627 Aminobenzoate degradation | 0.8 | 0.5 | 0.7 | 0.7 | 0.7 |
| ko00363 Bisphenol degradation | 0.5 | 0.3 | 0.4 | 0.4 | 0.4 |
| **Genetic Information Processing** | **10.0** | **10.6** | **9.7** | **10.1** | **10.1** |
| **Translation** | **4.1** | **4.3** | **4.0** | **4.1** | **4.1** |
| ko00970 Aminoacyl tRNA biosynthesis | 2.6 | 2.7 | 2.5 | 2.6 | 2.6 |
| ko03010 Ribosome | 1.5 | 1.6 | 1.5 | 1.5 | 1.5 |
| **Folding, sorting and degradation** | **1.7** | **2.0** | **1.6** | **1.8** | **1.8** |
| ko03018 RNA degradation | 1.1 | 1.4 | 1.1 | 1.2 | 1.2 |
| ko03060 Protein export | 0.6 | 0.6 | 0.5 | 0.6 | 0.6 |
| **Replication and repair** | **4.2** | **4.3** | **4.1** | **4.2** | **4.2** |
| ko03420 Nucleotide excision repair | 1.1 | 1.1 | 1.0 | 1.1 | 1.1 |
| ko03440 Homologous recombination | 1.1 | 1.0 | 1.1 | 1.1 | 1.1 |
| ko03430 Mismatch repair | 0.7 | 1.0 | 0.8 | 0.8 | 0.8 |
| ko03410 Base excision repair | 0.8 | 0.7 | 0.7 | 0.7 | 0.7 |
| ko03030 DNA replication | 0.5 | 0.5 | 0.5 | 0.5 | 0.5 |
| **Environmental Information Processing** | **15.0** | **17.2** | **18.0** | **17.3** | **17.7** |
| **Membrane transport** | **10.3** | **7.8** | **11.4** | **10.4** | **10.5** |
| ko02010 ABC transporters | 9.4 | 6.1 | 10.0 | 9.0 | 9.1 |
| ko03070 Bacterial secretion system | 0.9 | 1.7 | 1.4 | 1.4 | 1.4 |
| **Signal transduction** | **4.7** | **9.4** | **6.6** | **6.9** | **6.8** |
| ko02020 Two component system | 4.7 | 9.4 | 6.6 | 6.9 | 6.8 |
| **Cellular Processes** | **1.8** | **3.8** | **3.2** | **3.2** | **3.0** |
| **Cell growth and death** | **0.9** | **1.4** | **1.4** | **1.3** | **1.3** |
| ko04112 Cell cycle Caulobacter | 0.9 | 1.4 | 1.4 | 1.3 | 1.3 |
| **Cell motility** | **0.9** | **2.4** | **1.8** | **1.9** | **1.7** |
| ko02030 Bacterial chemotaxis | 0.5 | 1.2 | 0.9 | 1.0 | 0.9 |
| ko02040 Flagellar assembly | 0.4 | 1.2 | 0.9 | 0.9 | 0.8 |
